# Outpatient Teaching and Feedback Skills Workshop for Resident Physicians

**DOI:** 10.15766/mep_2374-8265.10930

**Published:** 2020-07-31

**Authors:** John Ryan Hayes, Jennifer Zeman, Bryan Johnston, Neal Campbell

**Affiliations:** 1 Assistant Professor, Department of Community and Family Medicine, Medical College of Wisconsin; 2 Resident, PGY 3, Department of Family Medicine, Waukesha Family Medicine Residency Program; 3 Resident, PGY 3, Department of Family Medicine, Columbia St. Mary's Family Medicine Residency Program; 4 Resident, PGY 3, Department of Family Medicine, All Saints Family Medicine Residency Program

**Keywords:** Residents as Teachers, Family Medicine, Teaching Workshop, Clinical Teaching, One-Minute Preceptor, Role-Play, RIME Model, ARCH Model, Feedback

## Abstract

**Introduction:**

There is a need for innovative workshops designed to teach students and residents the basics of clinical medical education. Resident physicians often spend a significant portion of their training teaching others and frequently have very little formal instruction on teaching techniques. Other teaching tools exist but are often either entirely lecture based or too extensive to easily incorporate into a residency teaching session. There is a need for the facilitated practice of teaching methods to improve the resident educational experience.

**Methods:**

This 80-minute workshop blends interactive role-play case studies with quick lectures on the ARCH feedback model, the RIME model of medical information mastery, and the One-Minute Preceptor. This workshop includes three short PowerPoint lectures, four case studies, a handout, a pre-/posttest, and a session evaluation form.

**Results:**

Resident and student learners were engaged for the entirety of this session. Pre-/posttest results showed an improvement in understanding of basic teaching and feedback techniques, and survey results showed a higher likelihood of the learners wanting to incorporate teaching into their future practice.

**Discussion:**

This workshop is quick and overall quite effective in teaching basic feedback and teaching techniques. It provides a much-needed opportunity for residents to practice teaching techniques immediately after they have learned the concepts. This training is ideal for a residency program looking to provide new senior residents with the teaching tools they need for success.

## Educational Objectives

By the end of this activity, learners will be able to:

1. Identify the clinical utility of each of the RIME, ARCH, and One-Minute Preceptor (OMP) feedback models.
2. Describe the steps of the RIME, ARCH, and OMP feedback models.
3. Apply RIME, ARCH, and OMP teaching models immediately in case-based role-play exercises.
4. Demonstrate improved comfort and attitude towards clinical teaching via a pre-/posttest feedback assessment.

## Introduction

Resident physicians often act as teachers. Resident physicians spend up to 25% of their time teaching junior residents and medical students.^1,2^ Higher-quality resident teaching has correlated with better student performance and a better opinion of the specialty in question.^3,4^ Studies have attributed one-third of student medical knowledge to resident teaching, and two-thirds of medical students felt that residents played an important role in their education.^5,6^ Recognizing the importance of quality resident teaching, both the Accreditation Council for Graduate Medical Education and the Liaison Committee on Medical Education require residents to be educated in teaching methods.^7,8^

Despite the critical and prevalent nature of resident teaching expectations, residents often do not feel ready for this role. To meet this need, the literature, including *MedEdPORTAL*, is rife with teaching and feedback methodologies intended for residents and junior faculty.^9–14^ It seems most resident-as-teacher programs are well liked by learners, but few curricula are presented with rigorously reproducible outcome data.^9^ Several systematic reviews of resident-as-teachers curricula exist, describing virtually every possible type of educational intervention.^10,11^ Systematic reviews of resident-as-teacher curricula have consistently demonstrated that a learner's self-assessed teaching behaviors and confidence in teaching improve.^9–11^ One review concluded that the One-Minute Preceptor (OMP) is the most effective methodology for clinical teaching skills.^10^ Another systematic review noted best practices in feedback delivery and concluded that training improves feedback skills, learner satisfaction with feedback, and feedback effectiveness.^12^ Several systematic reviews have noted the challenge of designing an effective resident-as-teacher curriculum that can be efficiently delivered to busy residents.^9,11,12^ There is a call for more workshop-based interventions that focus on teaching and feedback skills training instead of lectures on educational theory.^9,11,12^ Given the available evidence, teaching and feedback skill-building workshops can be effective interventions, bridging the confidence gap many residents experience when participating in medical education.

In this report, we describe a relatively quick (80-minute) teaching and skill-building workshop designed to meet the uniquely time-constrained educational needs of busy residency programs. Our group created engaging role-play exercises designed to both maintain the interests our learners and provide opportunities to build teaching skills through experiential learning. We chose simple assessment, teaching, and feedback methodologies that could be effectively presented in 5-minute lectures prior to our role-playing exercises. Available reviews of resident-as-teacher curricula have demonstrated the importance of revisiting teaching curricula over time.^10^ To create a lasting intervention, we chose the RIME model for assessment, the OMP for teaching, and ARCH for feedback. These three mnemonics were easy to remember and could easily be printed on a laminated white-coat card that our learners could reference in the future during real-world teaching. Role-play exercises were utilized so that a relatively large group of learners (30+) could pair off and practice these skills simultaneously. While other resident-as-teacher workshops do exist on *MedEdPORTAL*, none of them allow for practice on assessment, teaching, and feedback tools in less than 90 minutes.

## Methods

This session was introduced, facilitated, and designed by a family medicine attending physician with experience in clinic-based teaching and innovative lecture models. To promote a peer-learning environment, three senior residents were invited to present and cofacilitate this session. These third-year postgraduate (PGY 3) residents were identified by their program directors as having a strong interest and ability in medical education. Before the presentation, our senior resident presenters reviewed articles on the ARCH feedback model,^15^ RIME teaching/educational development model,^16^ and OMP model.^17^ PowerPoint presentations were designed to succinctly explain the three teaching methods/tools (Appendix A). Each PowerPoint explained the effectiveness of each teaching method, the steps of each teaching method, and an example of how to use each teaching method. Lecturers also designed four unique role-play case studies, allowing learners to practice and receive ARCH feedback on the RIME and OMP teaching methods immediately after they had learned the techniques. The resident and student learners who participated in this teaching workshop were not instructed to prepare before the session. Learners were fed lunch and provided with an ample supply of snacks and caffeinated beverages prior to the session.

This 80-minute teaching workshop presentation took place in March 2019 at the Family Medicine Cure Clinical Conference at the Medical College of Wisconsin (MCW). The presentation was given to 32 family medicine residents at first year postgraduate (PGY 1), second-year postgraduate (PGY 2), and PGY 3 levels from multiple family medicine programs across Southeast Wisconsin. Eight third-year medical students from MCW were also present. Presenters gave three PowerPoint presentations, each lasting less than 10 minutes, on ARCH, RIME, and the OMP (Appendix A). Following the RIME and OMP PowerPoint presentations, learners were asked to do two case-study activities in approximately 25 minutes. Learners were also supplied with a laminated pocket teaching card to serve as a reference as they completed these activities (Appendix B).

Prior to each role-play case-study session, learners were separated into pairs, assigned a role (student or preceptor), and given case-study handouts. Learners assigned to the student role were to read the prewritten case from the student's perspective. Learners assigned to the preceptor role were given the chief complaint and a teaching point/goal for the person in the student role to reach. At the conclusion of each case session, the preceptor used the ARCH model to provide feedback. Next, the learners were given another case study and instructed to switch roles; those who had played the student in the previous case were asked to play the preceptor and vice versa.

While learning the RIME model, our learners practiced the skill of pushing a student to the next level of learning. In our first case, a student presented the history of the present illness and lab values and then stopped. In the following case, the student presented a differential and then stopped. These cases were specifically written to teach a new educator not to take over and provide the answer to a clinical scenario. See Appendix C for the RIME cases.

For our OMP role-playing session, we designed cases that taught our preceptors how to instruct and provide feedback for difficult learners. In our first scenario, the learner provided an excellent history and a detailed physical, proposed an elaborate plan, but ignored the social history so that the plan was entirely unrealistic for the patient. The learner was also instructed to provide a very positive self-assessment although having clearly missed the point. In our second scenario, the learner was instructed to miss several concerning medical cues and misdiagnose a patient. This learner was told to provide an overwhelmingly negative self-assessment. Precepting these common mistakes in clinical reasoning and self-assessment helped our learners apply the core concepts of the ARCH and OMP models. See Appendix D for the OMP cases.

To determine baseline knowledge and assess for improvement, audience members were asked to complete a pre- and posttest (Appendix E) that included multiple-choice questions on the teaching tools presented. A self-assessment of comfort level when teaching during busy clinic days was handed out and completed both before and after the presentation. Following the presentation, overall feedback forms were completed.

## Results

Of the 32 resident attendees, all 32 completed the postlecture evaluation, and 31 completed the pre- and postquiz as described above. Results are summarized in Tables 1–4. Table 1 shows the responses to pre- and postquiz prompts about willingness to teach learners and indicates that an increased number of respondents were willing to teach learners after the workshop. Table 2 shows the percentage of correct responses to the three multiple-choice content questions that were included in the pre- and postquiz. Participants were also given the chance to leave comments on strengths and weaknesses of the workshop. These comments were reviewed for common themes, which are presented in Table 3. Four common themes were identified: the interactivity of the workshop, improved feedback-giving techniques, the pocket card that was handed out, and specific cases for each feedback model. Comments supporting each theme are presented in the table as well. Finally, Table 4 shows participants' overall ratings of the workshop and content. These prompts were evaluated using a 7-point scale (1 = *poor*, 3–4 = *average*, and 7 = *excellent*). Overall, the mean value for all three prompts, which asked about the presenters and the content, was over 6, with standard deviations from 0.55 to 0.62.

**Table 1.**
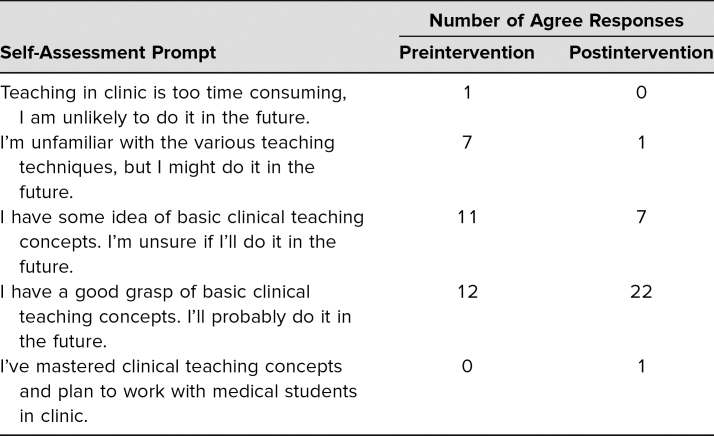
Number of Agree Responses to Pre- and Postquiz Survey About Willingness and Knowledge of Teaching Learners

**Table 2.**
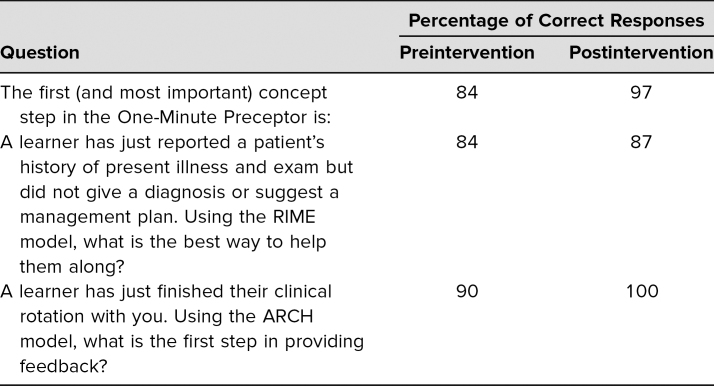
Percentage of Correct Answers to Questions About the Three Learning Models

**Table 3.**
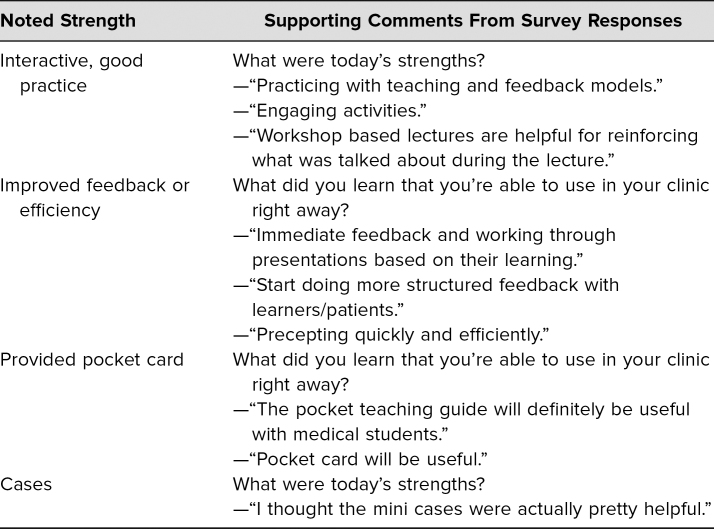
Summary of Participants' Postworkshop Comments Grouped by Main Themes

**Table 4.**
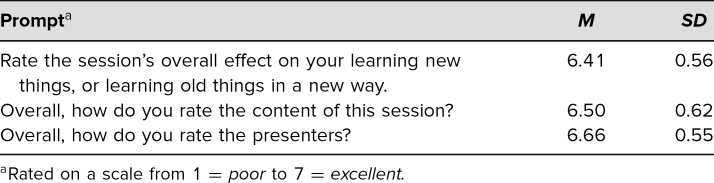
Evaluation of Presentation by Participants (*N* = 32)

## Discussion

To address the need for acceptable resident teaching education in our residency programs—in particular, the intervention had to be time efficient, outpatient focused, and immediately applicable—we developed and implemented a brief intervention exposing residents to established teaching models with the opportunity to immediately practice these skills in a simulated environment.

Too often in residency training, brand-new senior PGY 2 residents are underprepared to take on the role of managing interns and medical students. This teaching workshop provides an efficient mechanism to orient residents and medical students to participating in medical education. In 80 minutes, with no preparation required, this session effectively trains learners on the ARCH feedback model, the OMP clinical teaching model, and the RIME model for medical learner development. Instead of focusing on lectures to accomplish our learning goals, 50 of the 80 minutes in our session are dedicated to facilitated role-play, allowing our learners to practice the skills they have just learned. At the conclusion of our session, learners reported almost unanimous positive feedback and demonstrated pre-/posttest knowledge gain.

Implementation was approached in a team-based manner, with a faculty member guiding vision and structure and resident teaching leaders from separate but related programs recruited to collaboratively create curricula for their peers. Evaluating the curricula of three separate but related programs for resident teacher training was an initial challenge, as exposure to teacher training was found to be uncoordinated and somewhat disjointed. Curricular gatekeepers were interviewed at the various institutions, who all expressed the perceived need for a teacher training intervention program created within the above constraints, while also assisting in the identification of resident teaching leaders suitable for program involvement. Scheduling challenges were an additional difficulty in preparation and implementation, as resident and faculty schedules were constrained, making group meetings difficult. Time frames were planned far in advance, and electronic collaboration was utilized to mitigate this barrier. Didactic scheduling was another constraint, with CURE Conference time slots reserved far in advance and highly competitive given that they represented an opportunity to reach many residents at multiple programs. This scarcity of didactic time contributed to the decision to pursue a single session for this intervention. Evaluation design and implementation were determined to be best approached in a simple pre-/posttest knowledge and attitude assessment. A more robust evaluation process was not pursued due to time and resource constraints.

During the course itself, learners were observed to be engaged, particularly during the role-play case components. Certain less-polished features of the cases, such as the dramatic reaction of one learner upon receiving formative feedback (“I wonder if I have what it takes to be a doctor”), were observed to invite disbelief and pushed some learners away from the intended learning objectives. Observations such as these were noted and used to revisit and improve the case studies.

Limitations were several. Because this is a one-off intervention, long-term impact expectations are modest. Learners would likely benefit from a reinforcement of these teaching skills and further education introducing additional teaching approaches in an ongoing manner. Evaluation did not include longitudinal knowledge or attitude change, behavior change, or stimulation of continued self-learning in teaching methods. These areas were deemed to be outside the scope of this intervention's objectives and were also viewed as unfeasible given lack of available time and resources. Cases were not vetted by the learner audience prior to the intervention, which could have improved the experience.

This teaching workshop is a useful tool for enabling learners to act as more effective outpatient medical educators. By the time learners have progressed to the latter half of medical school and residency, they have considerable experience in medical education. At this point, students and residents have their own teaching styles and opinions. Facilitated role-play allows them to be individuals, improving upon their educational identities while following the general guidelines set forth. This training is capable of providing an effective teaching tool to residency programs with similar goals and constraints.

## Appendices

ARCH, RIME, and OMP Training Materials.pptxPocket Teaching Guide.docxRIME Role-Play Case Studies.docxOMP Role-Play Case Studies.docxPre- and Posttest.docx All appendices are peer reviewed as integral parts of the Original Publication.
