# Supplementary material for: Outpatient Teaching and Feedback Skills Workshop for Resident Physicians
Source: MedEdPORTAL. 2020 Jul 31;16:10930. doi: 10.15766/mep_2374-8265.10930 (PMC7394347; doi:10.15766/mep_2374-8265.10930)
Supplement: Supplementary file 1 — ARCH, RIME, and OMP Training Materials.pptxPocket Teaching Guide.docxRIME Role-Play Case Studies.docxOMP Role-Play Case Studies.docxPre- and Posttest.docx [file mep_2374-8265.10930-s001.zip › A. ARCH, RIME, and OMP Training Materials.pptx]

## Slide 1
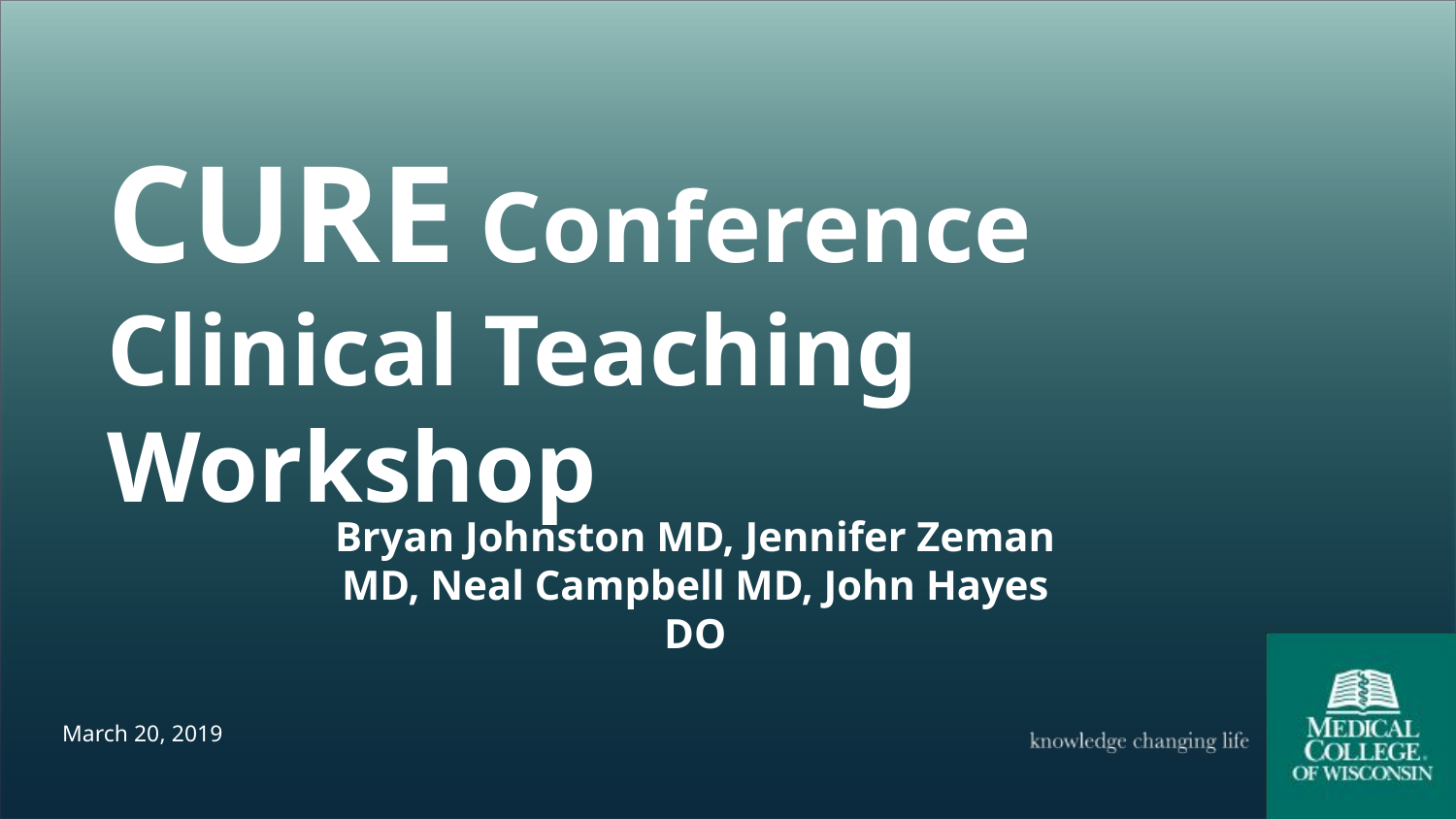

# CURE Conference Clinical Teaching Workshop
Bryan Johnston MD, Jennifer Zeman MD, Neal Campbell MD, John Hayes DO
March 20, 2019

## Slide 2
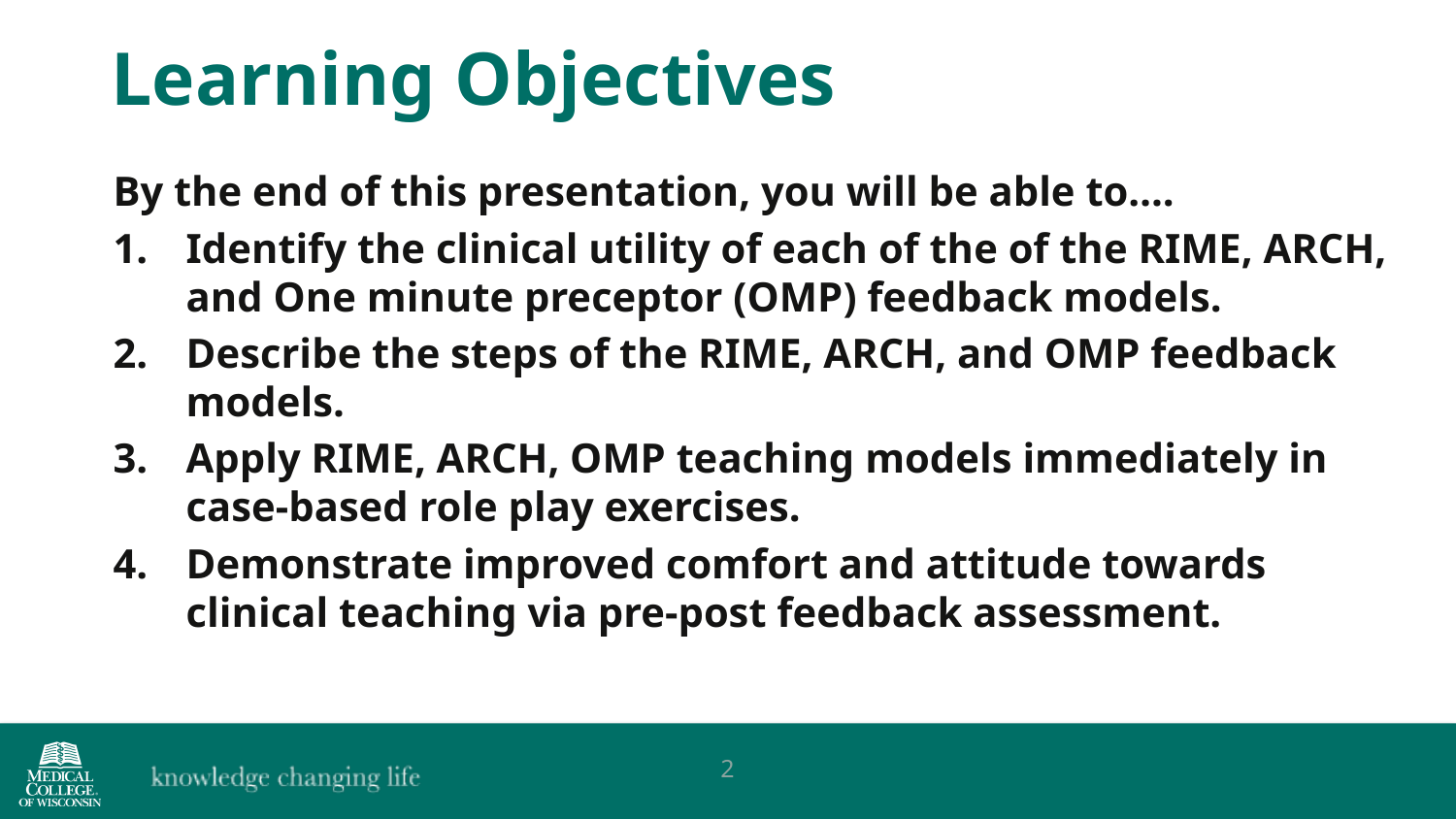

Learning Objectives
By the end of this presentation, you will be able to….
Identify the clinical utility of each of the of the RIME, ARCH, and One minute preceptor (OMP) feedback models.
Describe the steps of the RIME, ARCH, and OMP feedback models.
Apply RIME, ARCH, OMP teaching models immediately in case-based role play exercises.
Demonstrate improved comfort and attitude towards clinical teaching via pre-post feedback assessment.
2

## Slide 3
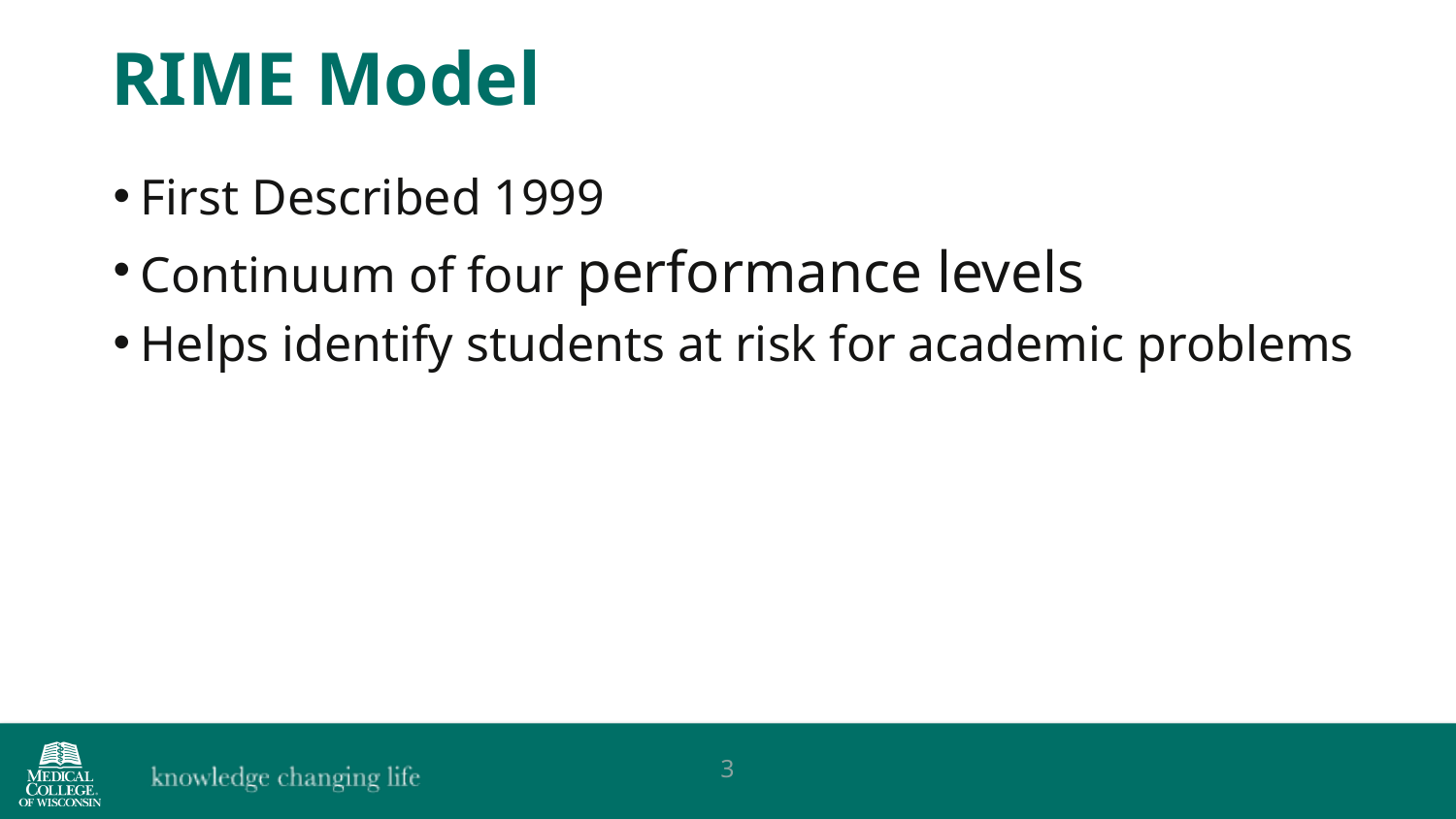

RIME Model
First Described 1999
Continuum of four performance levels
Helps identify students at risk for academic problems
3

## Slide 4
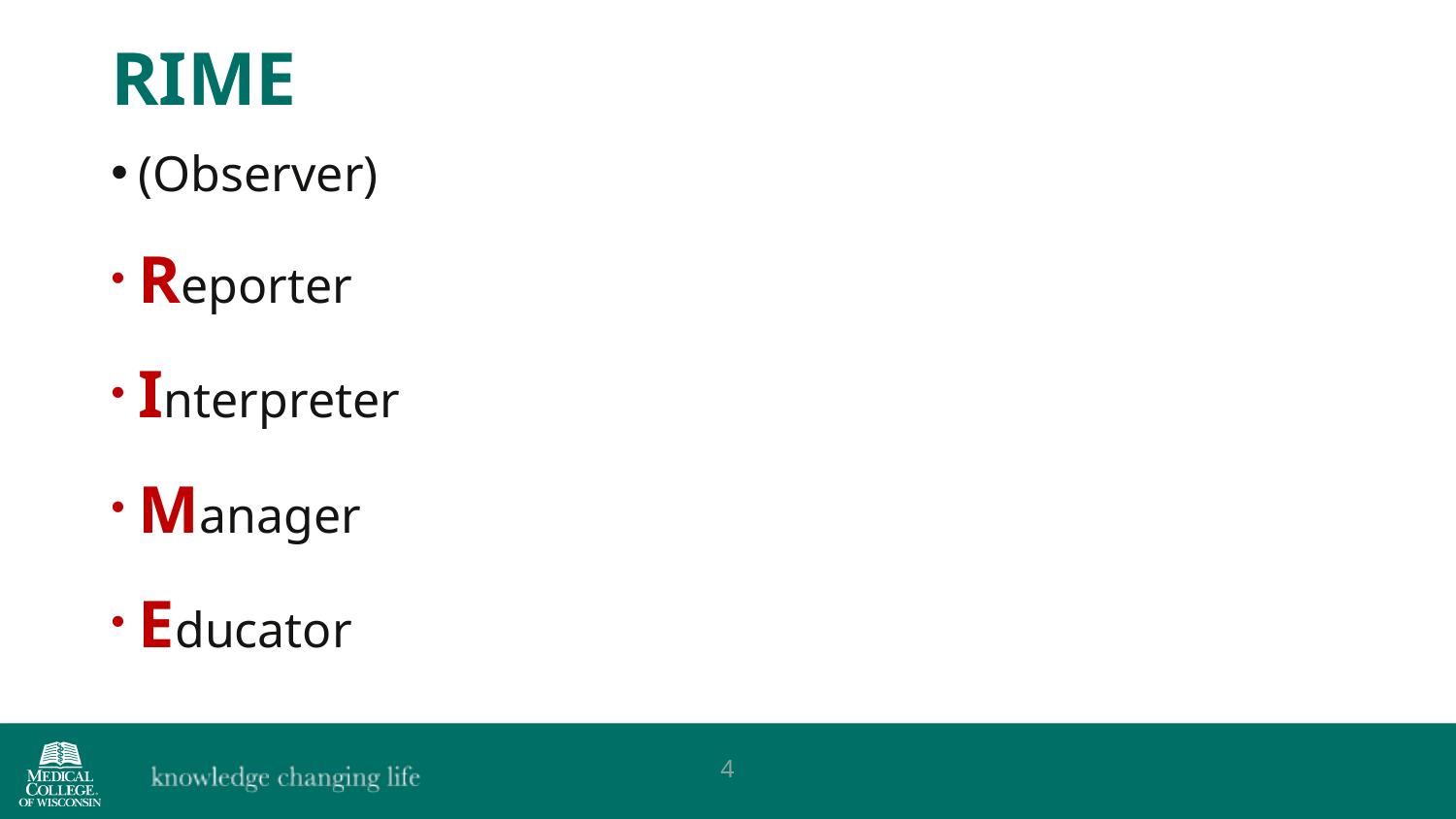

RIME
(Observer)
Reporter
Interpreter
Manager
Educator
4

## Slide 5
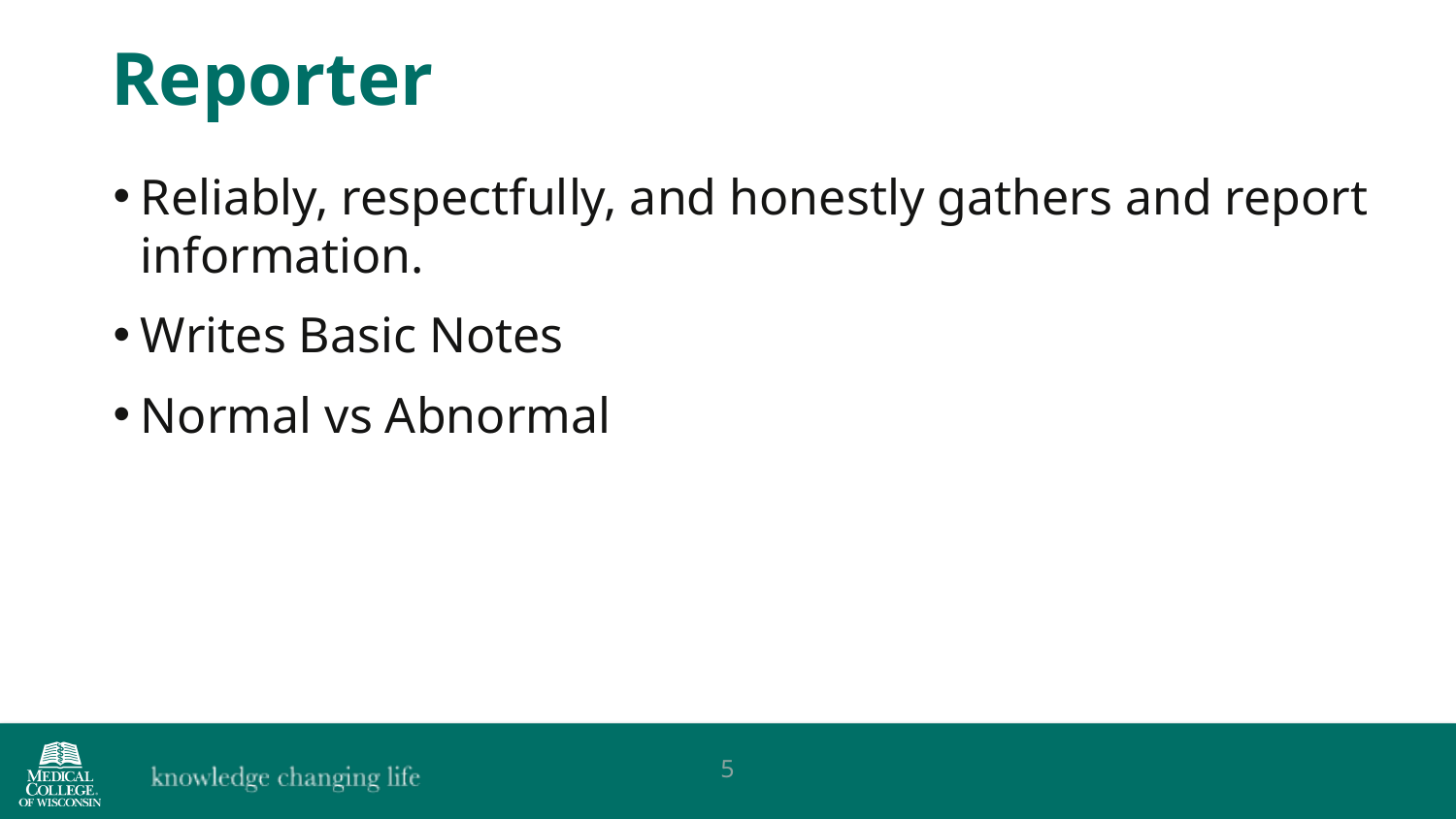

Reporter
Reliably, respectfully, and honestly gathers and report information.
Writes Basic Notes
Normal vs Abnormal
5

## Slide 6
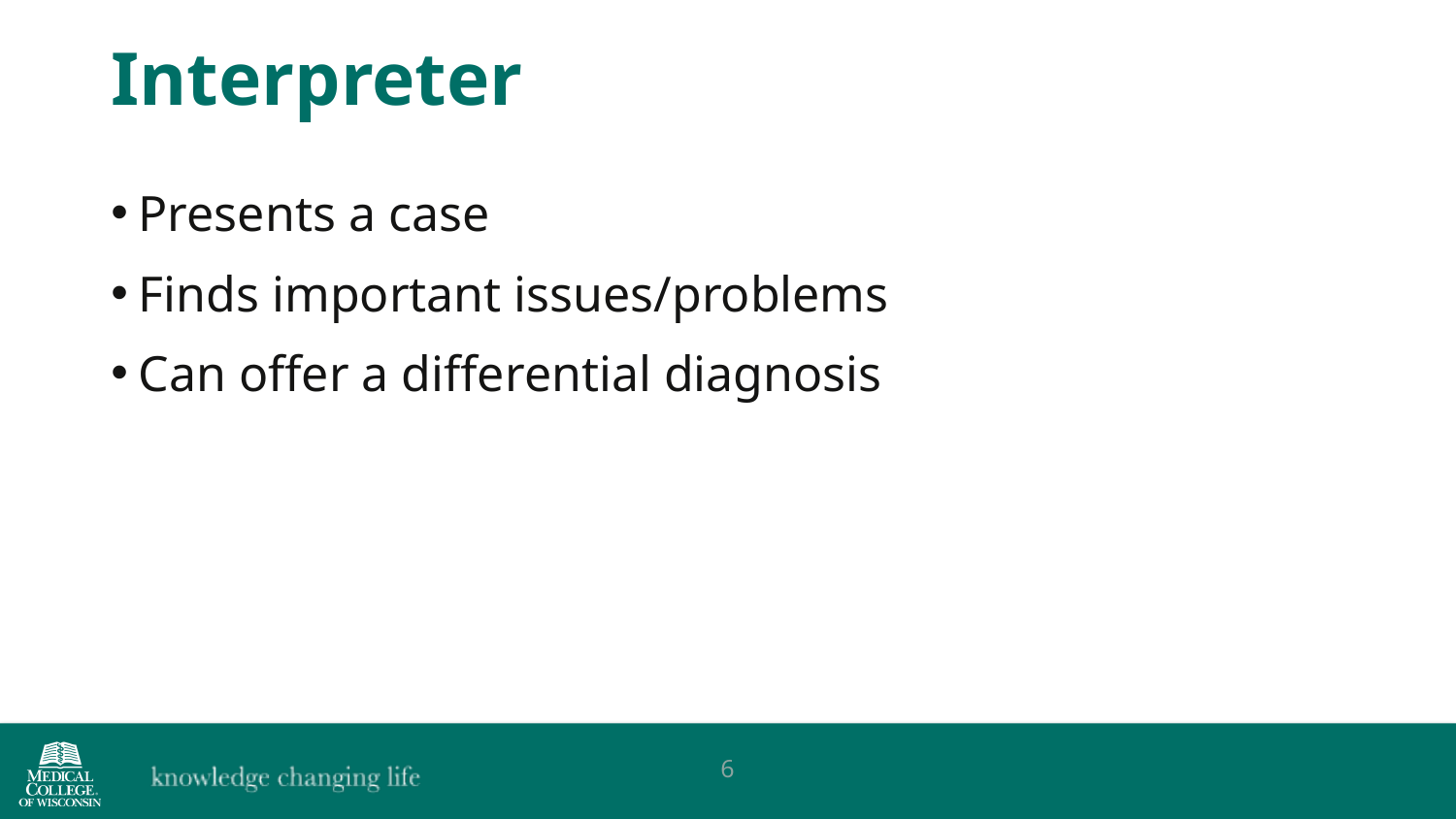

Interpreter
Presents a case
Finds important issues/problems
Can offer a differential diagnosis
6

## Slide 7
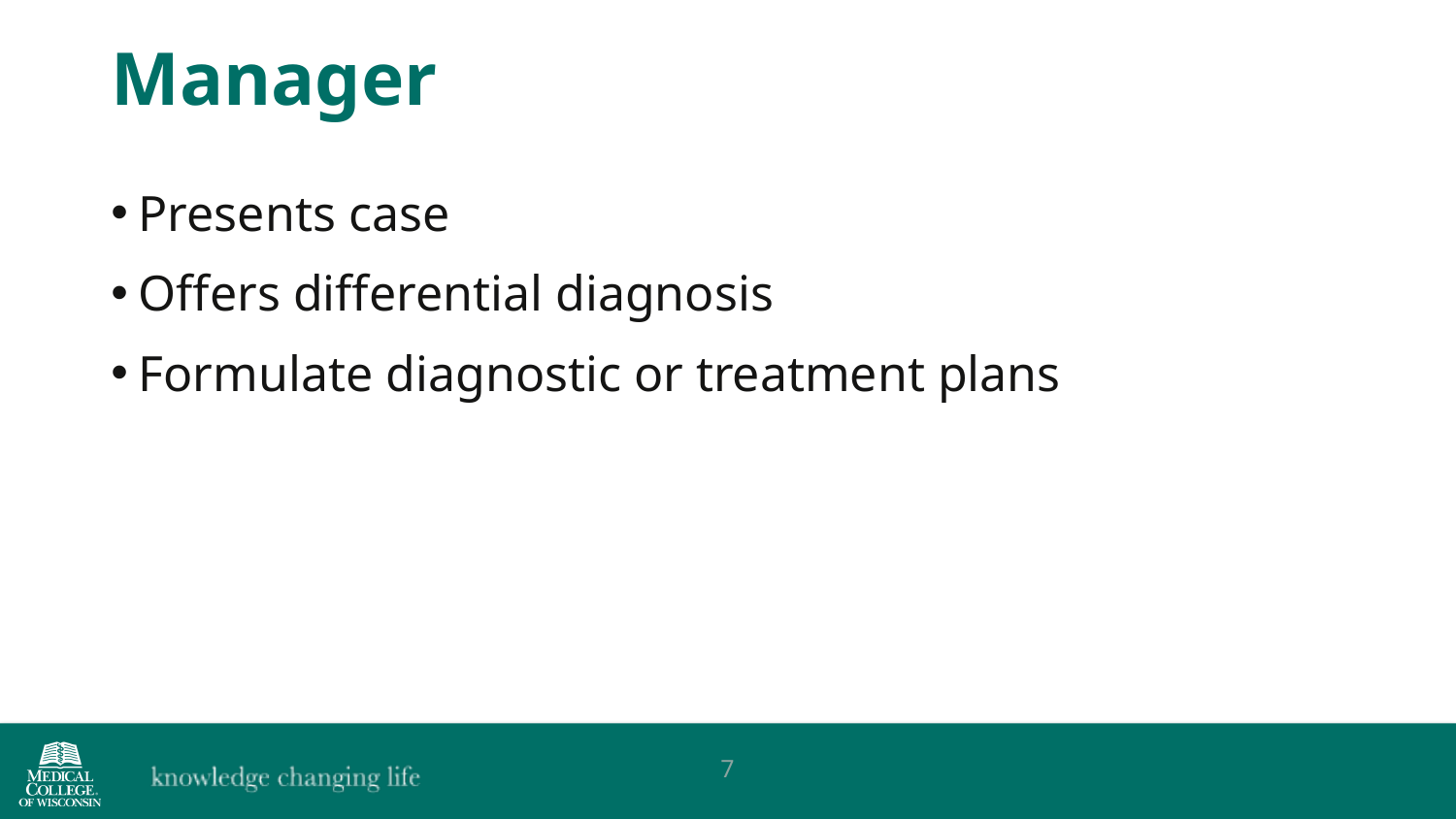

Manager
Presents case
Offers differential diagnosis
Formulate diagnostic or treatment plans
7

## Slide 8
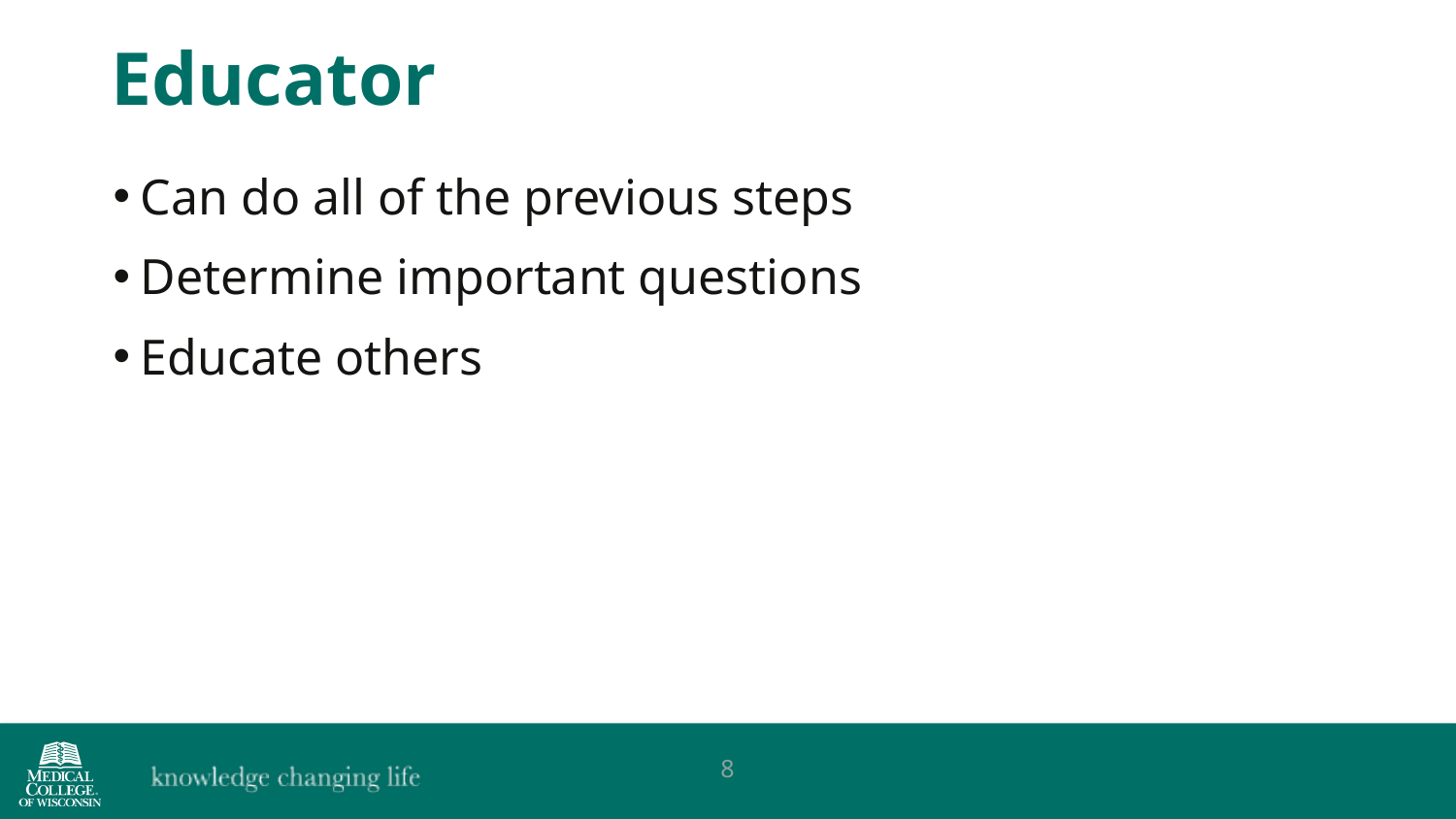

Educator
Can do all of the previous steps
Determine important questions
Educate others
8

## Slide 9
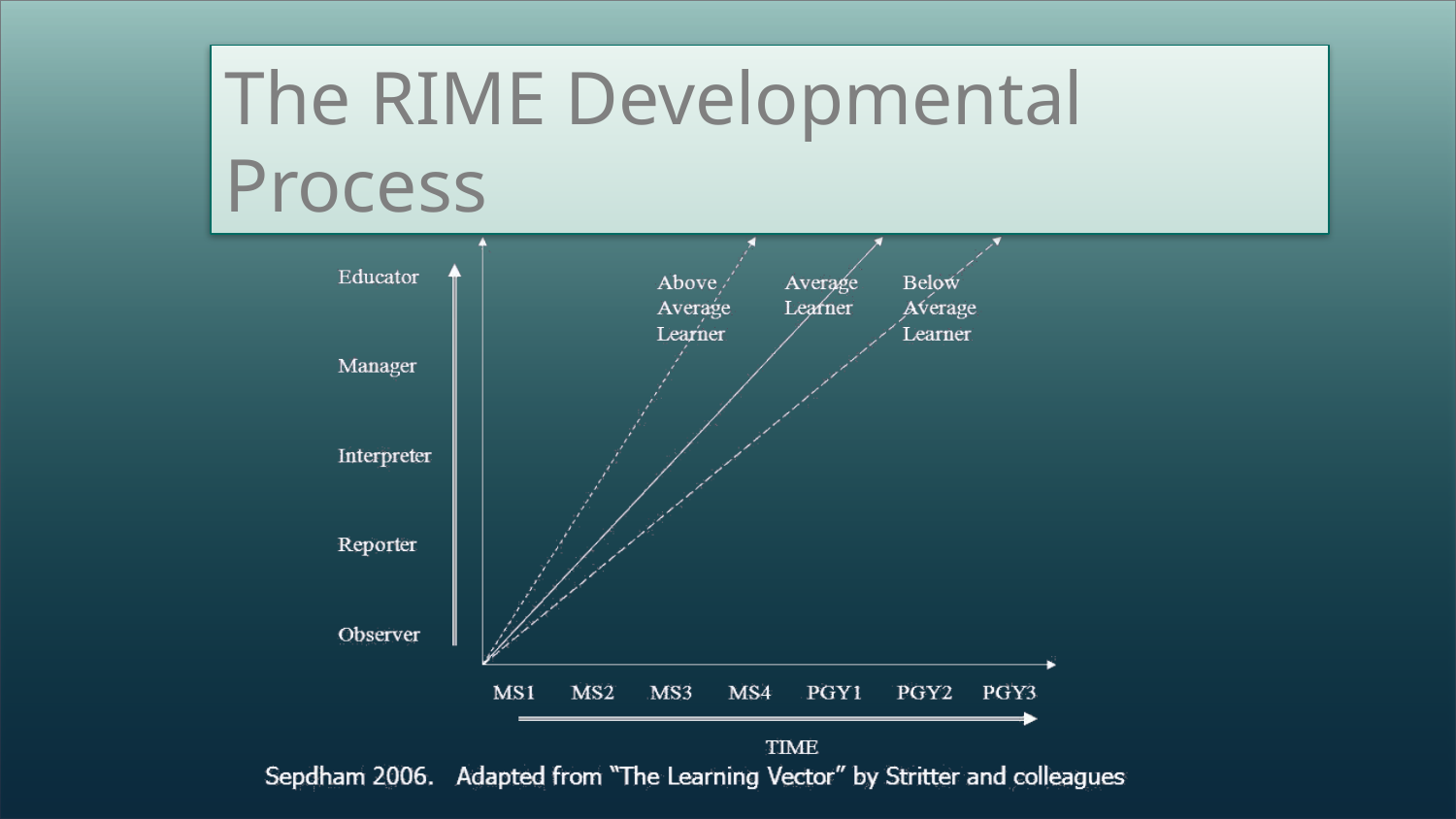

The RIME Developmental Process

## Slide 10
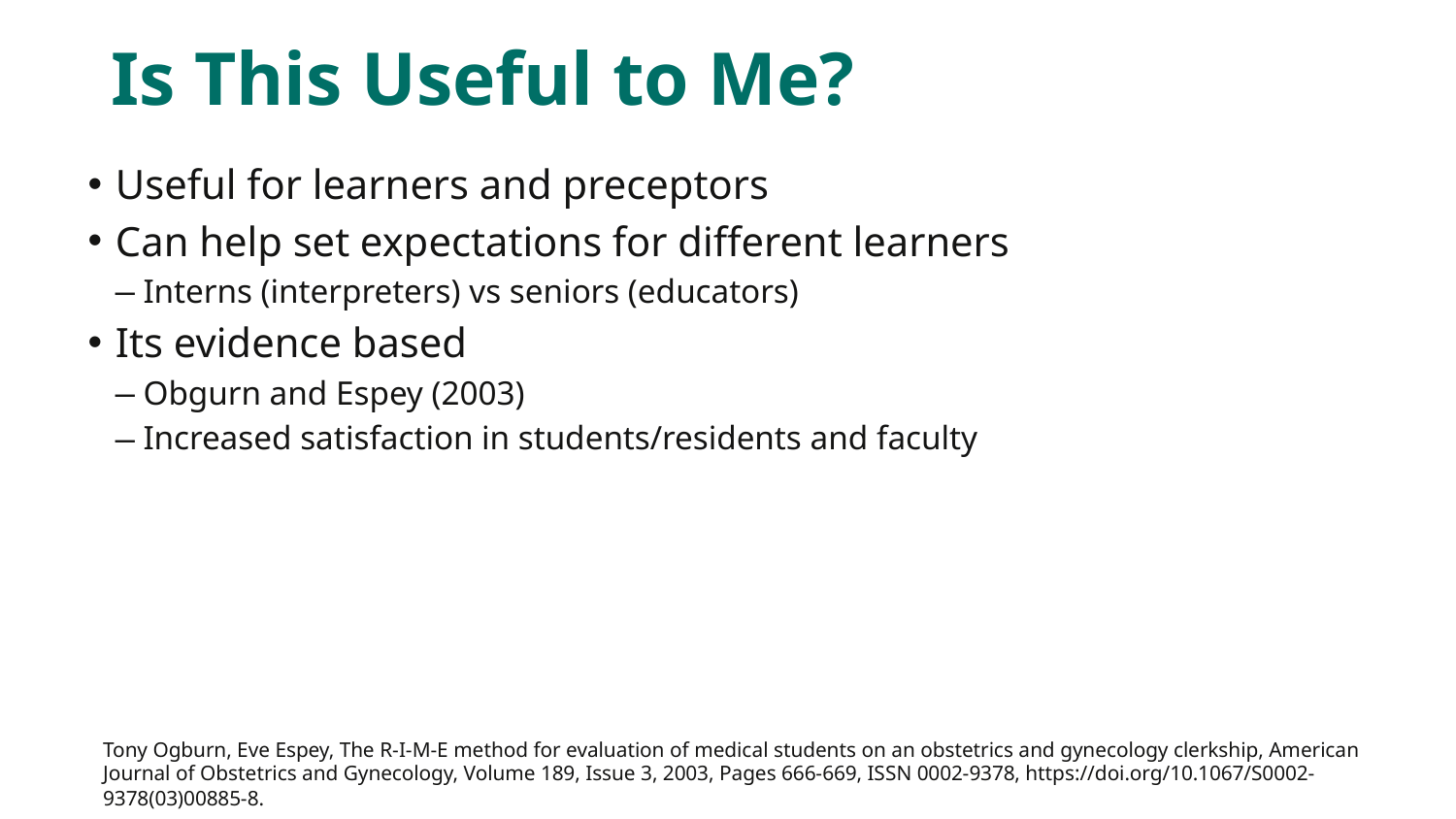

Is This Useful to Me?
Useful for learners and preceptors
Can help set expectations for different learners
Interns (interpreters) vs seniors (educators)
Its evidence based
Obgurn and Espey (2003)
Increased satisfaction in students/residents and faculty
Tony Ogburn, Eve Espey, The R-I-M-E method for evaluation of medical students on an obstetrics and gynecology clerkship, American Journal of Obstetrics and Gynecology, Volume 189, Issue 3, 2003, Pages 666-669, ISSN 0002-9378, https://doi.org/10.1067/S0002-9378(03)00885-8.

## Slide 11
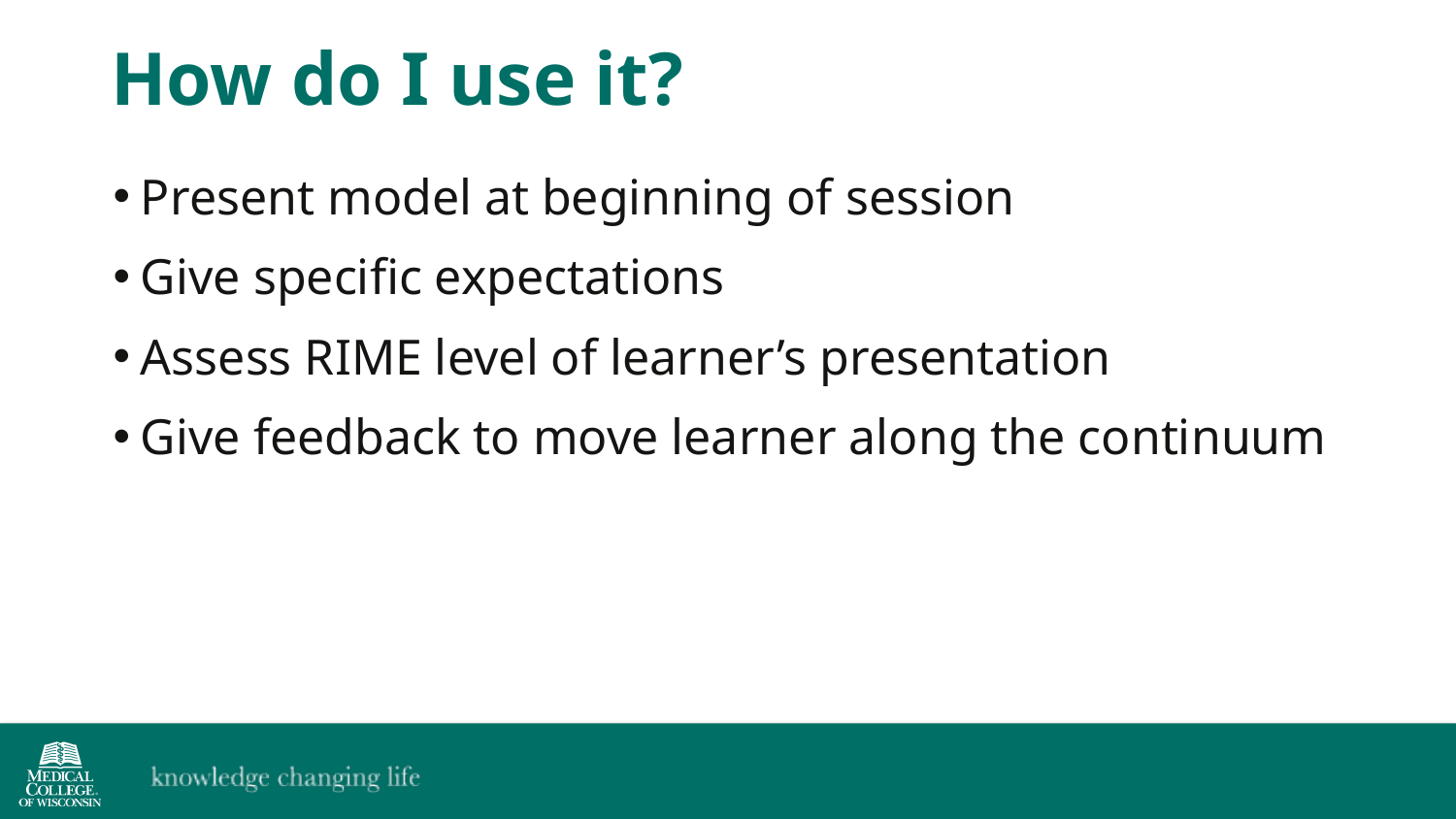

How do I use it?
Present model at beginning of session
Give specific expectations
Assess RIME level of learner’s presentation
Give feedback to move learner along the continuum

## Slide 12
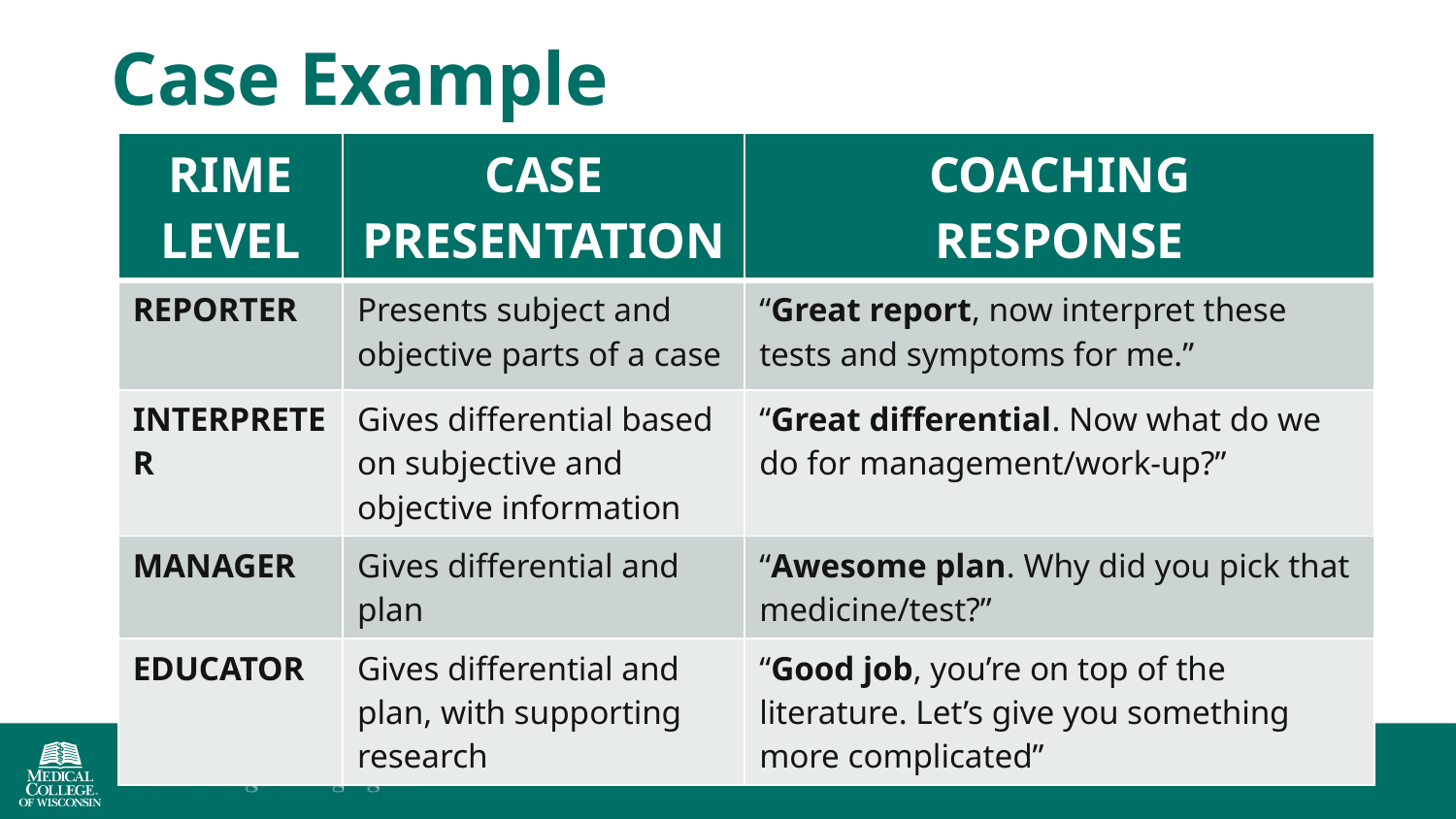

Case Example
| RIME level | Case Presentation | Coaching Response |
| --- | --- | --- |
| Reporter | Presents subject and objective parts of a case | “Great report, now interpret these tests and symptoms for me.” |
| Interpreter | Gives differential based on subjective and objective information | “Great differential. Now what do we do for management/work-up?” |
| Manager | Gives differential and plan | “Awesome plan. Why did you pick that medicine/test?” |
| Educator | Gives differential and plan, with supporting research | “Good job, you’re on top of the literature. Let’s give you something more complicated” |

## Slide 13
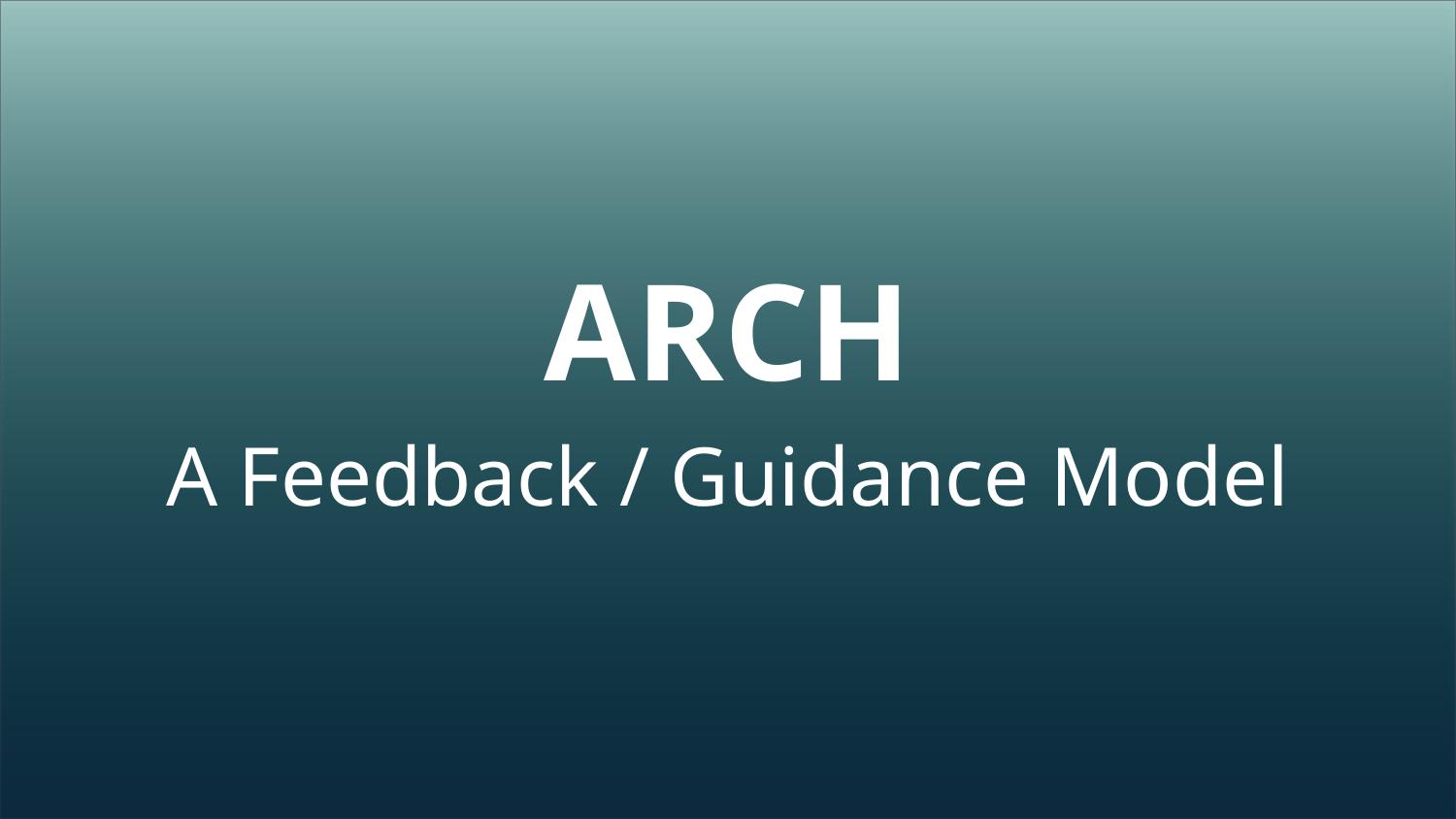

# ARCH A Feedback / Guidance Model

## Slide 14
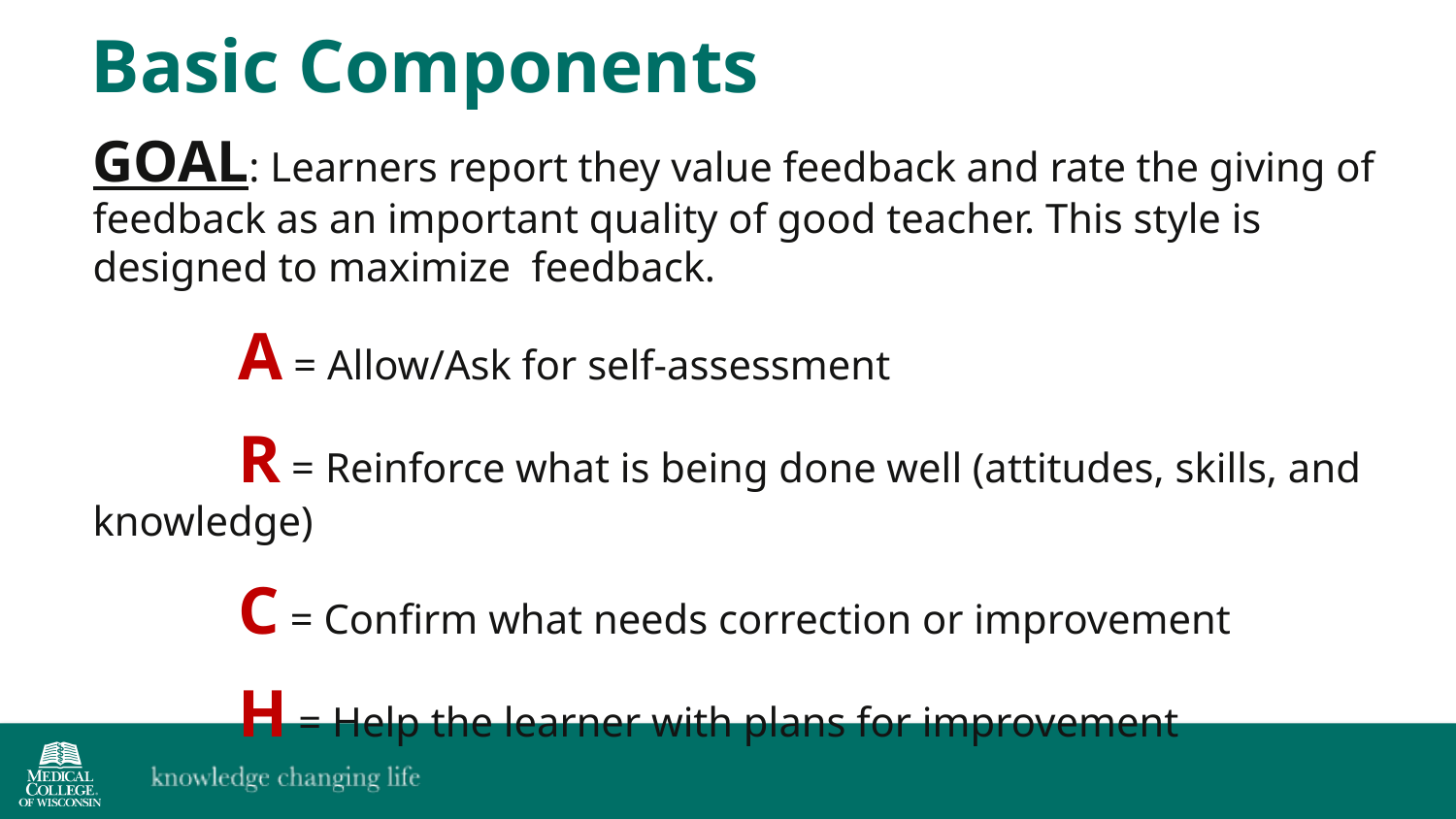

Basic Components
GOAL: Learners report they value feedback and rate the giving of feedback as an important quality of good teacher. This style is designed to maximize feedback.
	A = Allow/Ask for self-assessment
	R = Reinforce what is being done well (attitudes, skills, and knowledge)
	C = Confirm what needs correction or improvement
	H = Help the learner with plans for improvement

## Slide 15
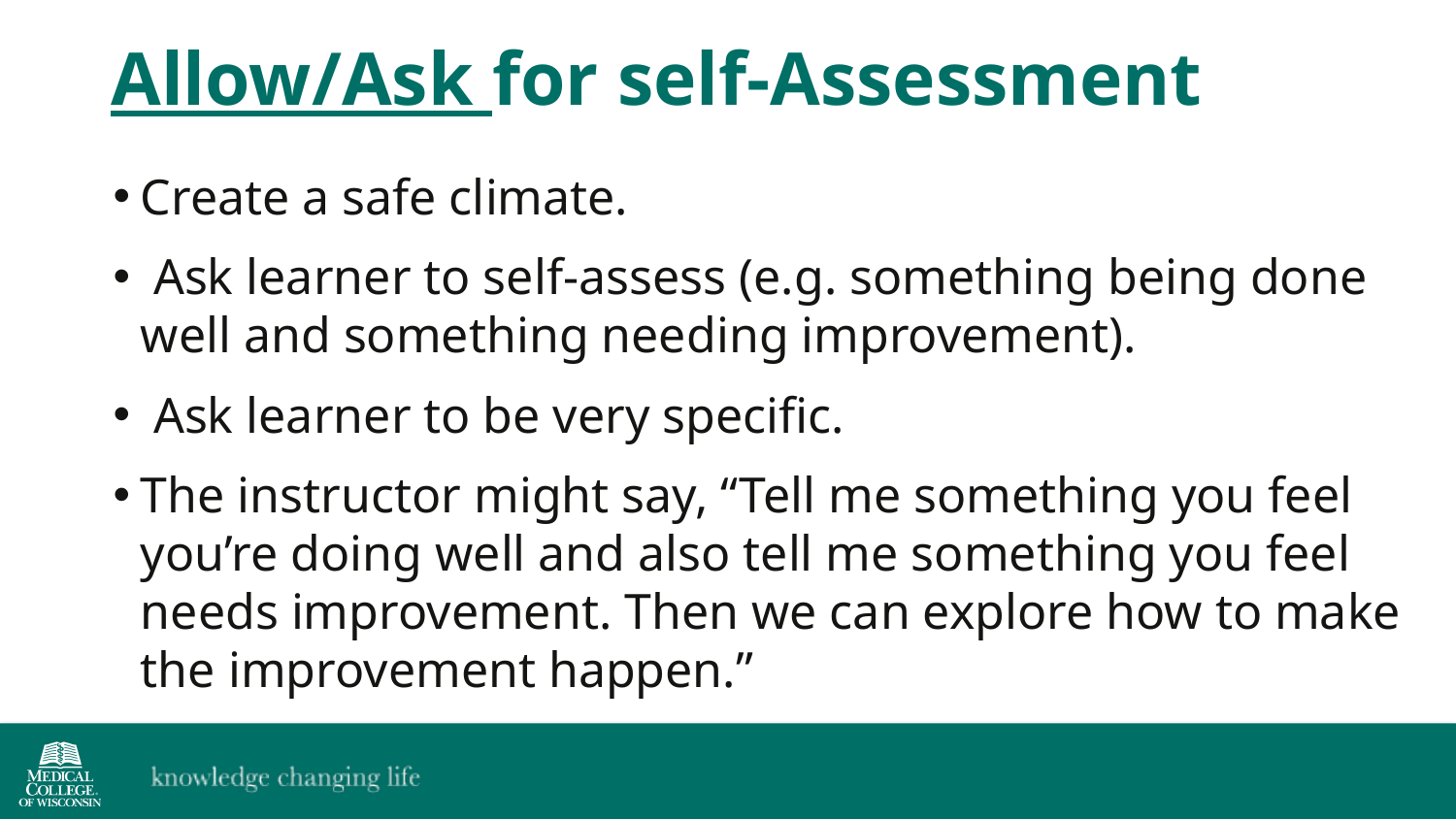

Allow/Ask for self-Assessment
Create a safe climate.
 Ask learner to self-assess (e.g. something being done well and something needing improvement).
 Ask learner to be very specific.
The instructor might say, “Tell me something you feel you’re doing well and also tell me something you feel needs improvement. Then we can explore how to make the improvement happen.”

## Slide 16
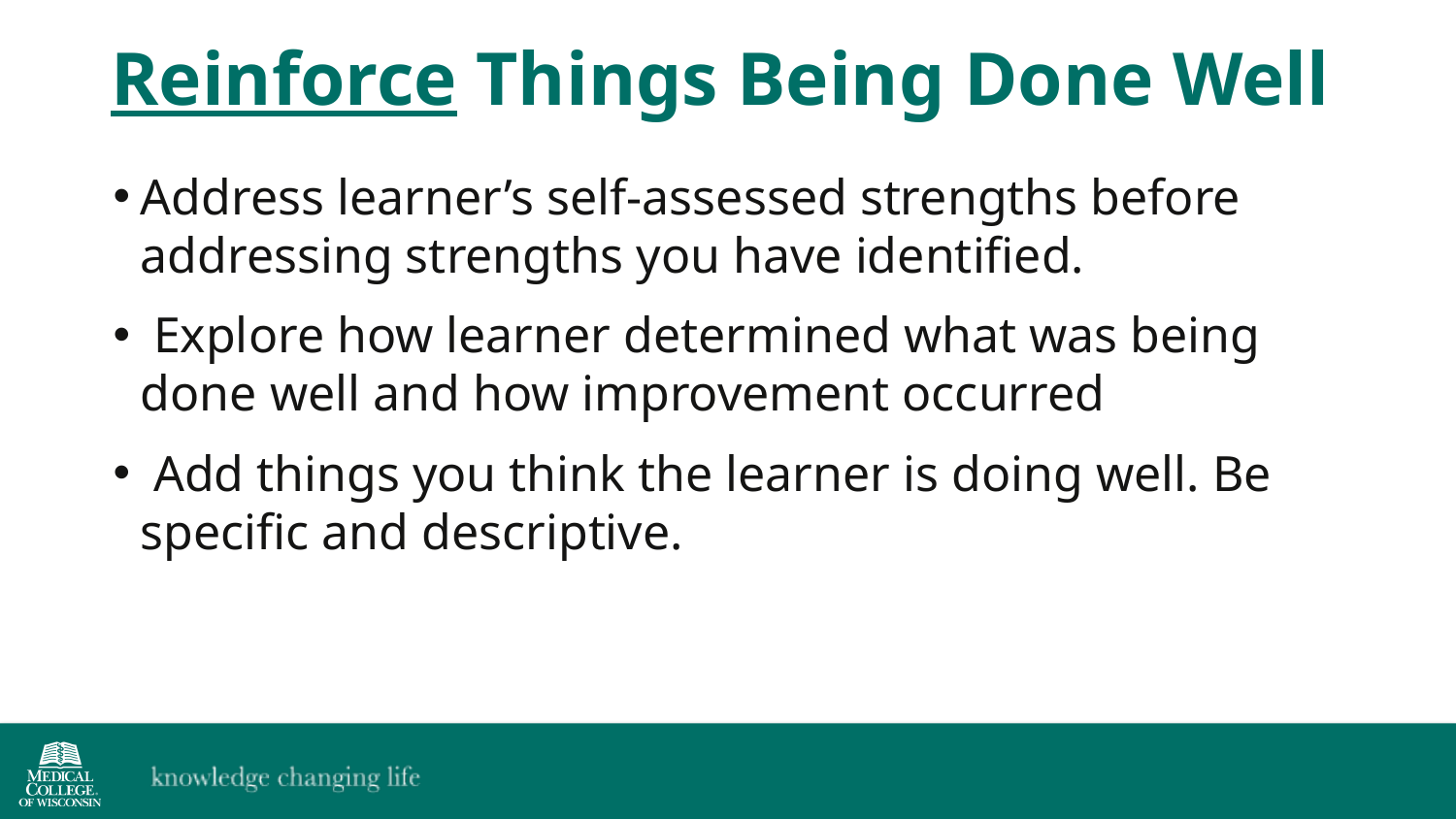

Reinforce Things Being Done Well
Address learner’s self-assessed strengths before addressing strengths you have identified.
 Explore how learner determined what was being done well and how improvement occurred
 Add things you think the learner is doing well. Be specific and descriptive.

## Slide 17
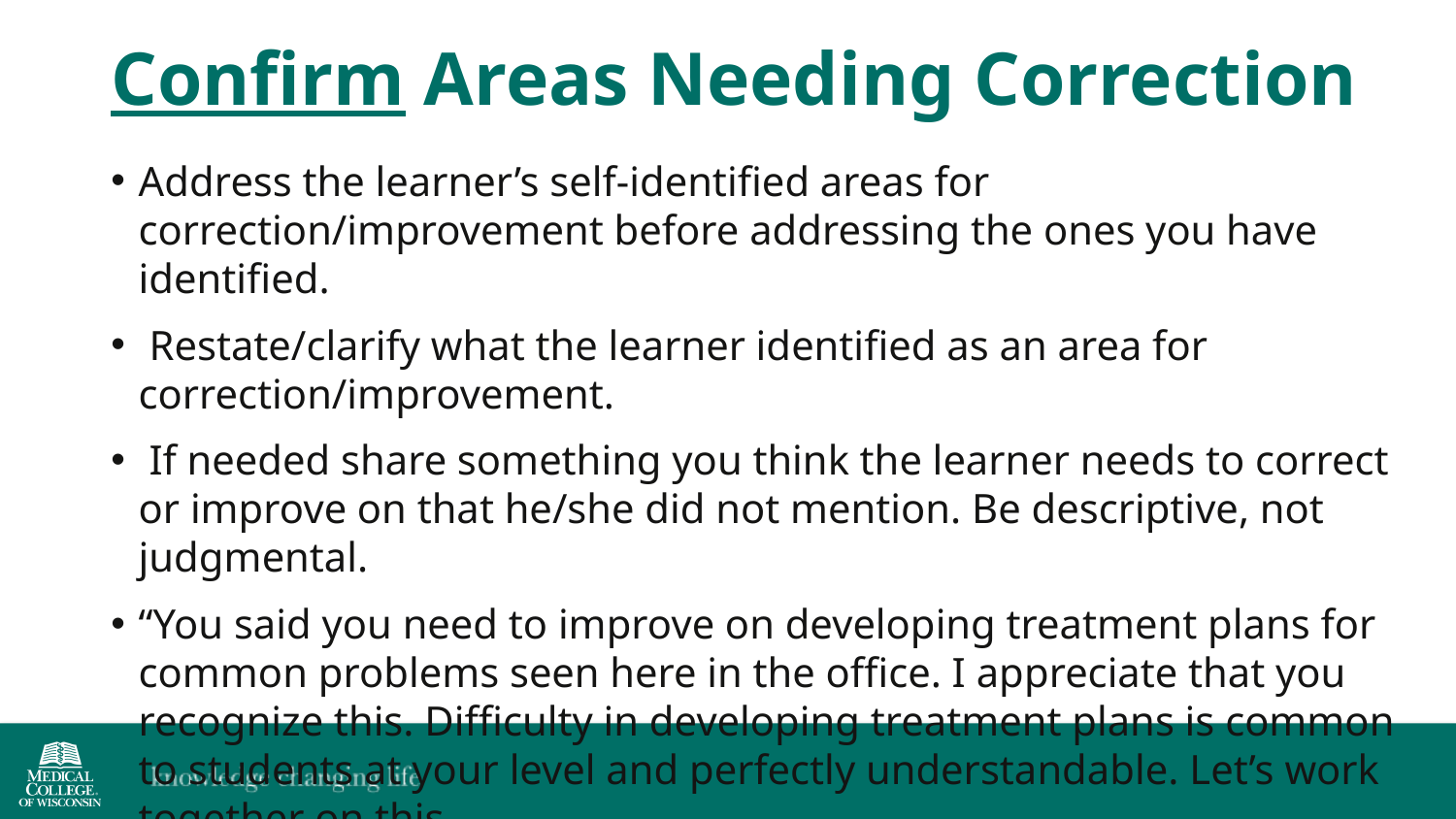

Confirm Areas Needing Correction
Address the learner’s self-identified areas for correction/improvement before addressing the ones you have identified.
 Restate/clarify what the learner identified as an area for correction/improvement.
 If needed share something you think the learner needs to correct or improve on that he/she did not mention. Be descriptive, not judgmental.
“You said you need to improve on developing treatment plans for common problems seen here in the office. I appreciate that you recognize this. Difficulty in developing treatment plans is common to students at your level and perfectly understandable. Let’s work together on this.

## Slide 18
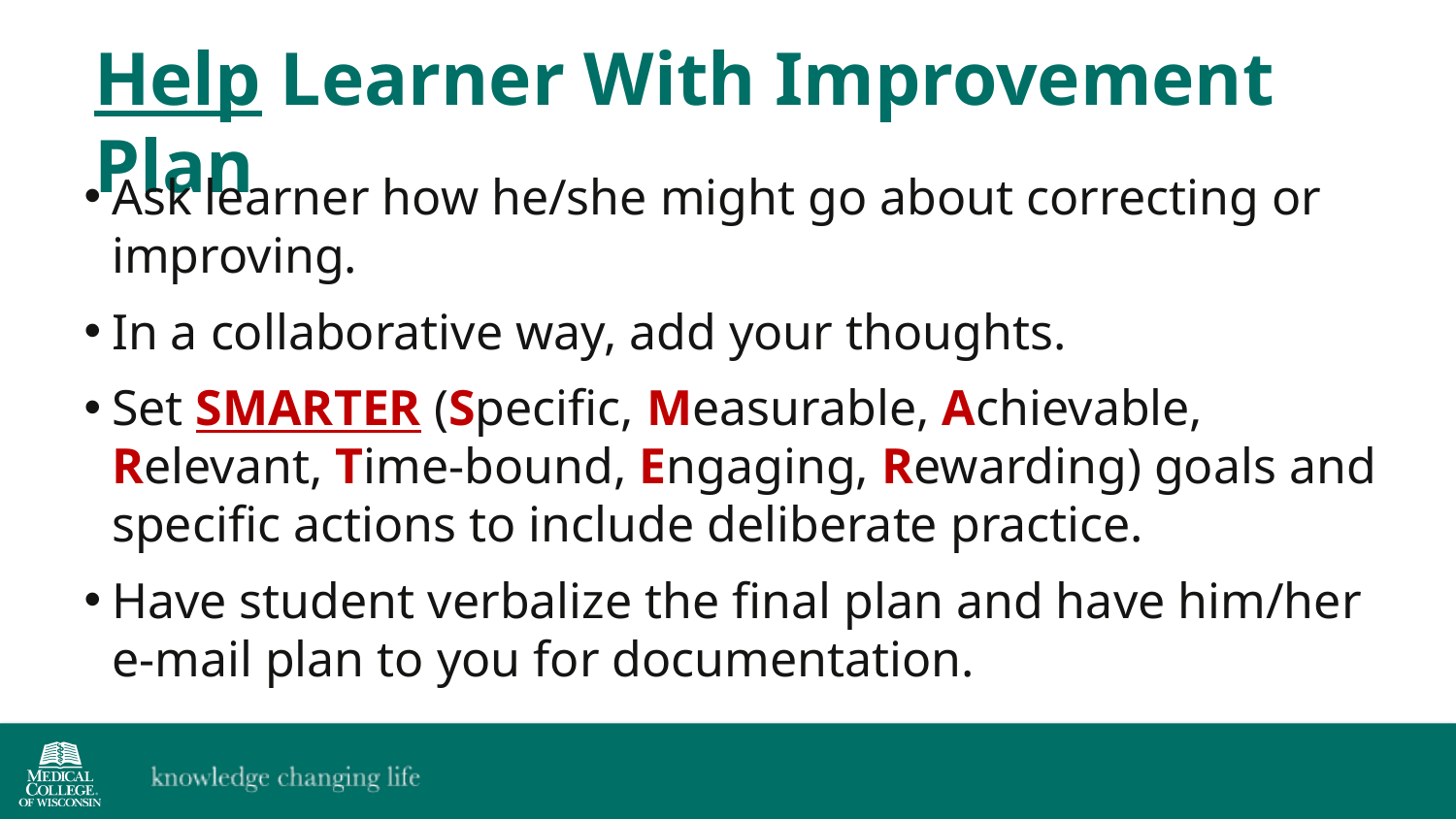

Help Learner With Improvement Plan
Ask learner how he/she might go about correcting or improving.
In a collaborative way, add your thoughts.
Set SMARTER (Specific, Measurable, Achievable, Relevant, Time-bound, Engaging, Rewarding) goals and specific actions to include deliberate practice.
Have student verbalize the final plan and have him/her e-mail plan to you for documentation.

## Slide 19
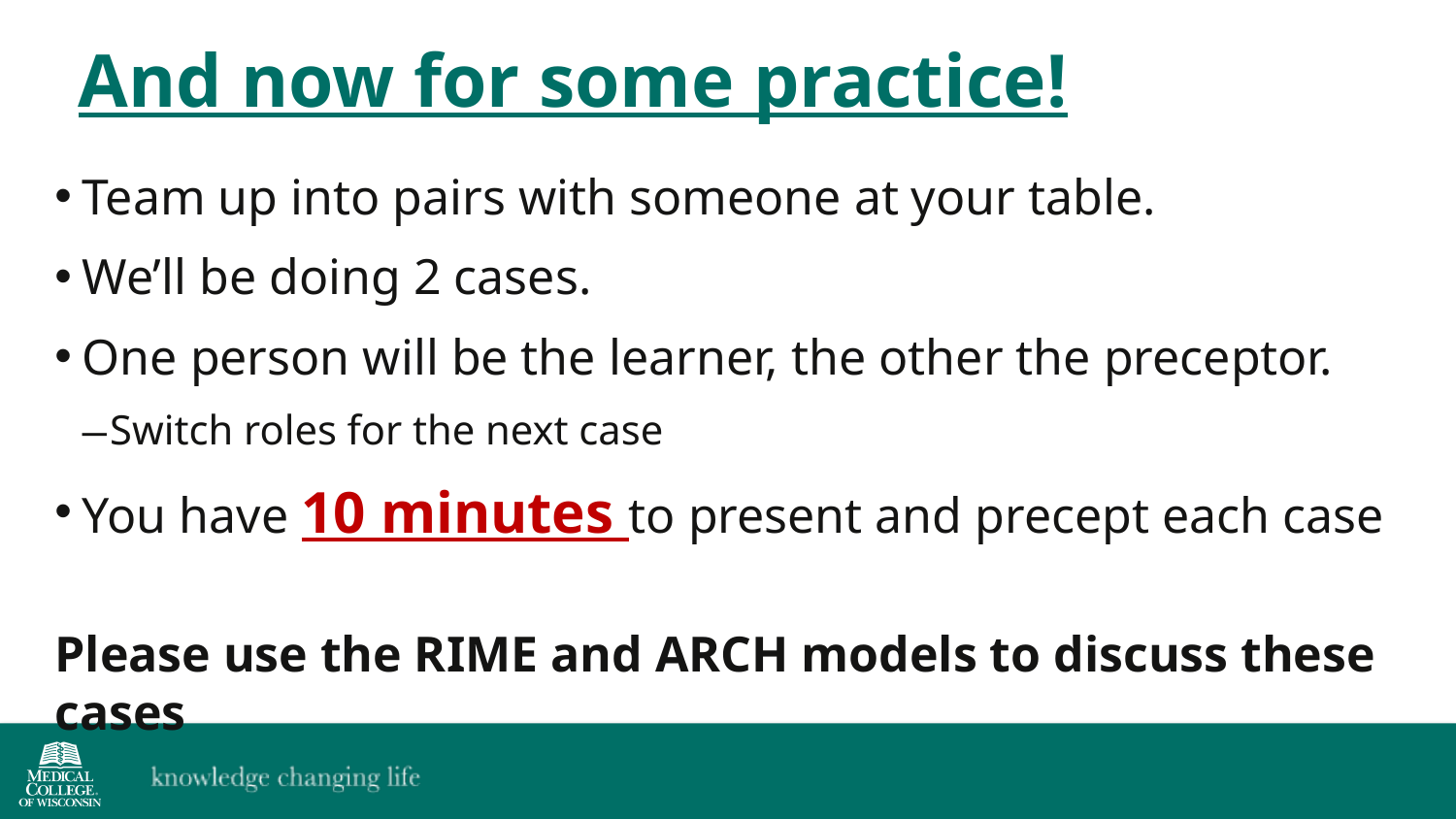

And now for some practice!
Team up into pairs with someone at your table.
We’ll be doing 2 cases.
One person will be the learner, the other the preceptor.
Switch roles for the next case
You have 10 minutes to present and precept each case
Please use the RIME and ARCH models to discuss these cases

## Slide 20
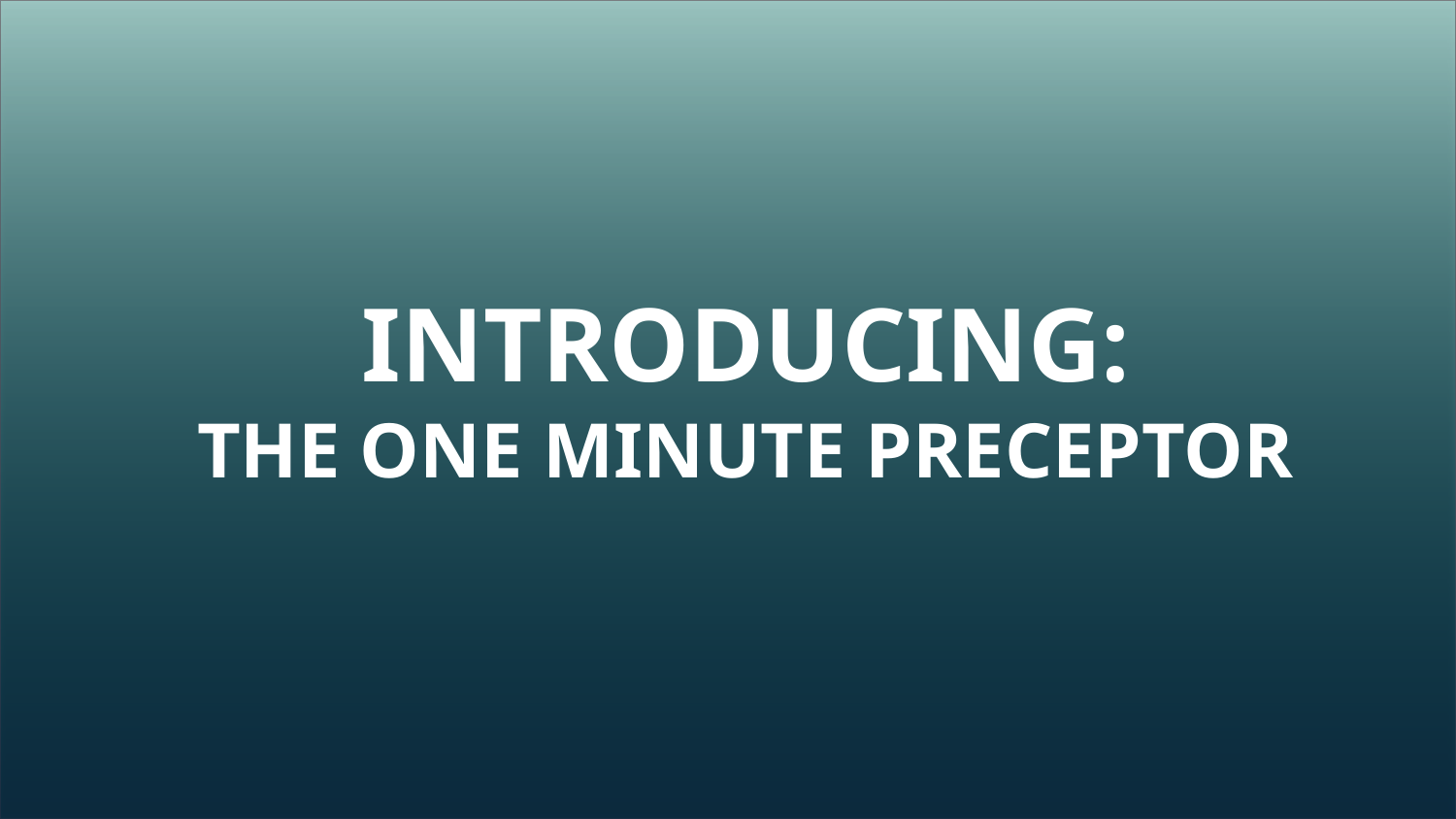

# INTRODUCING: THE One Minute Preceptor

## Slide 21
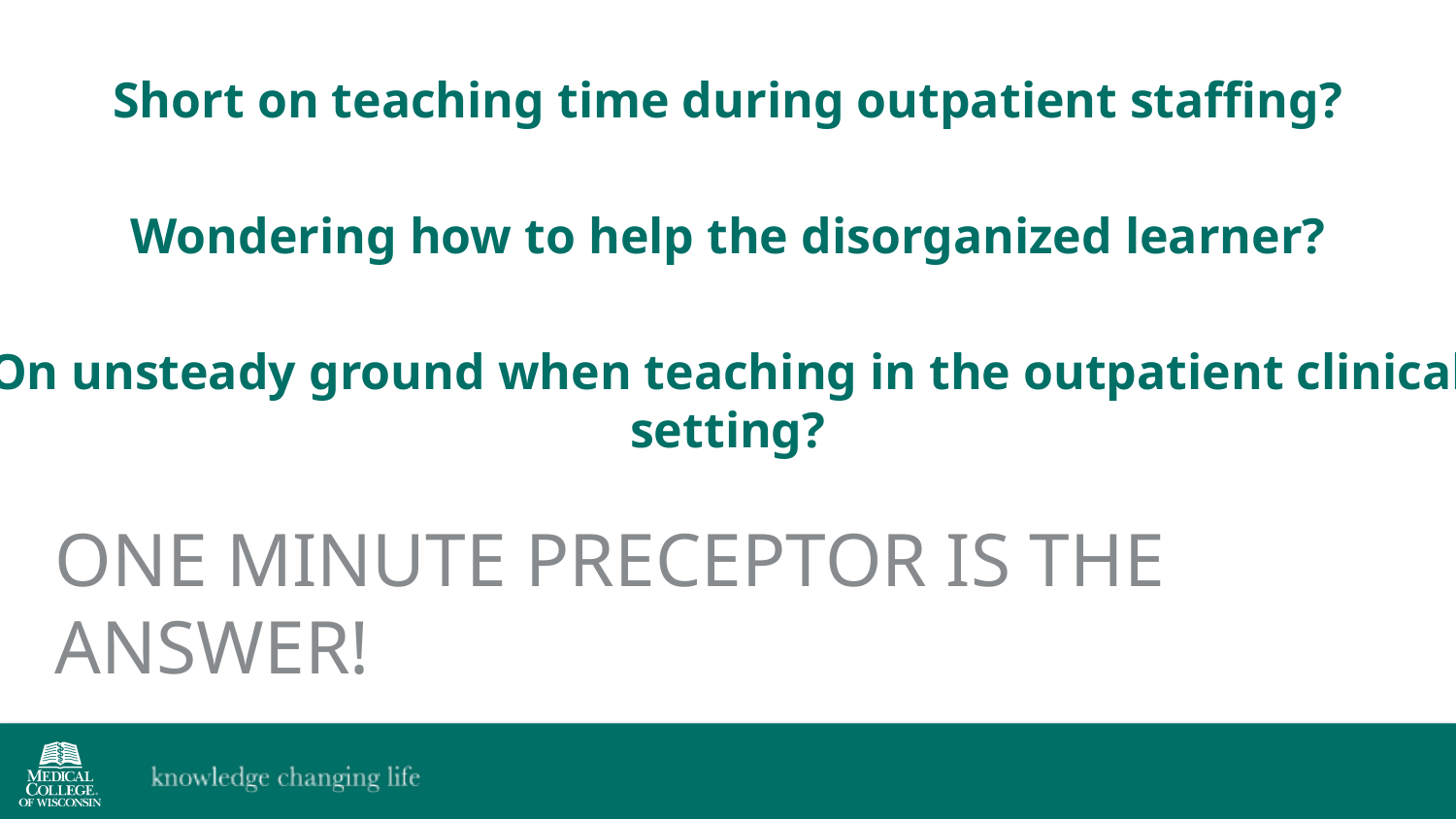

Short on teaching time during outpatient staffing?
Wondering how to help the disorganized learner?
On unsteady ground when teaching in the outpatient clinical setting?
ONE MINUTE PRECEPTOR IS THE ANSWER!

## Slide 22
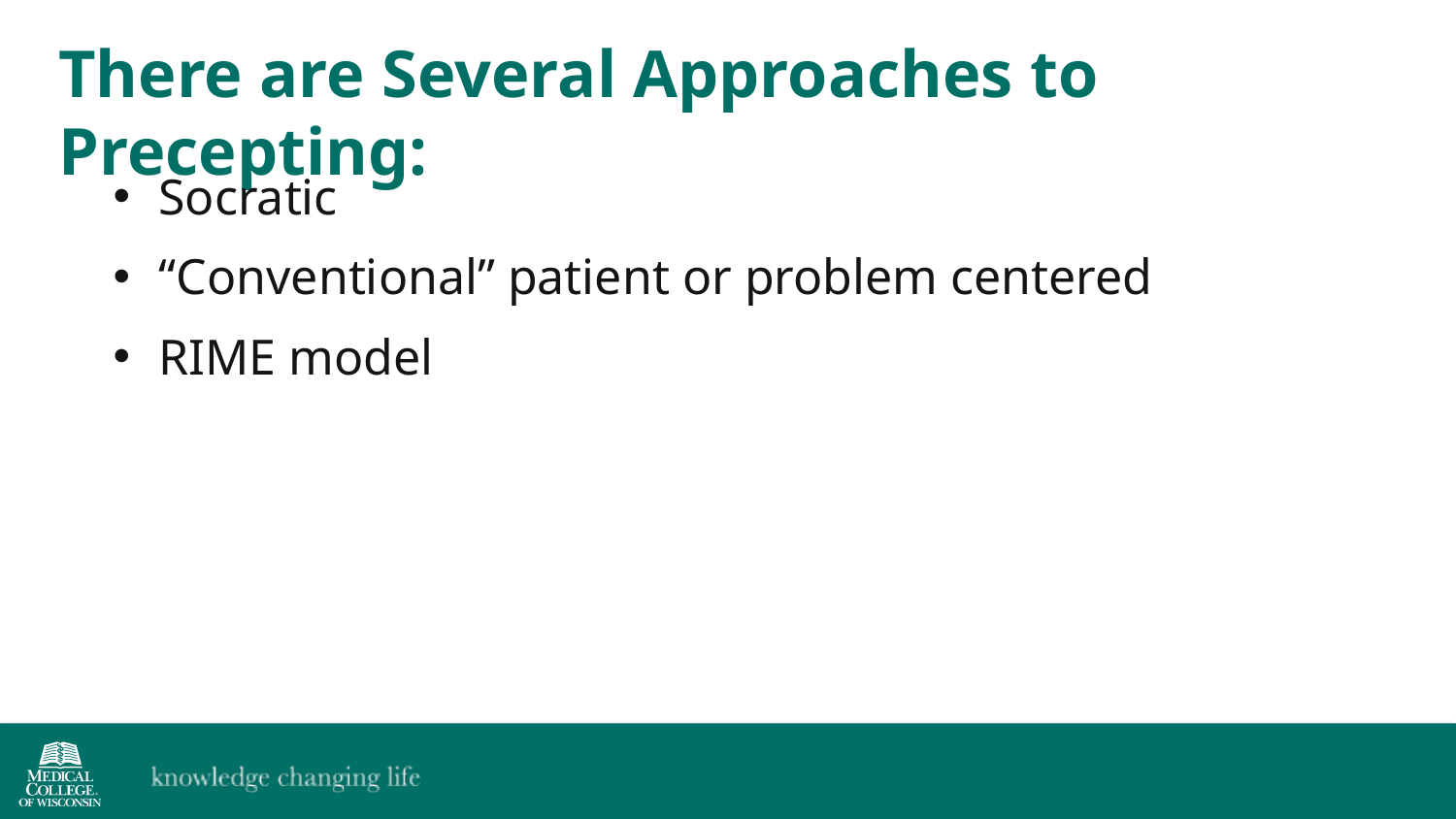

There are Several Approaches to Precepting:
Socratic
“Conventional” patient or problem centered
RIME model

## Slide 23
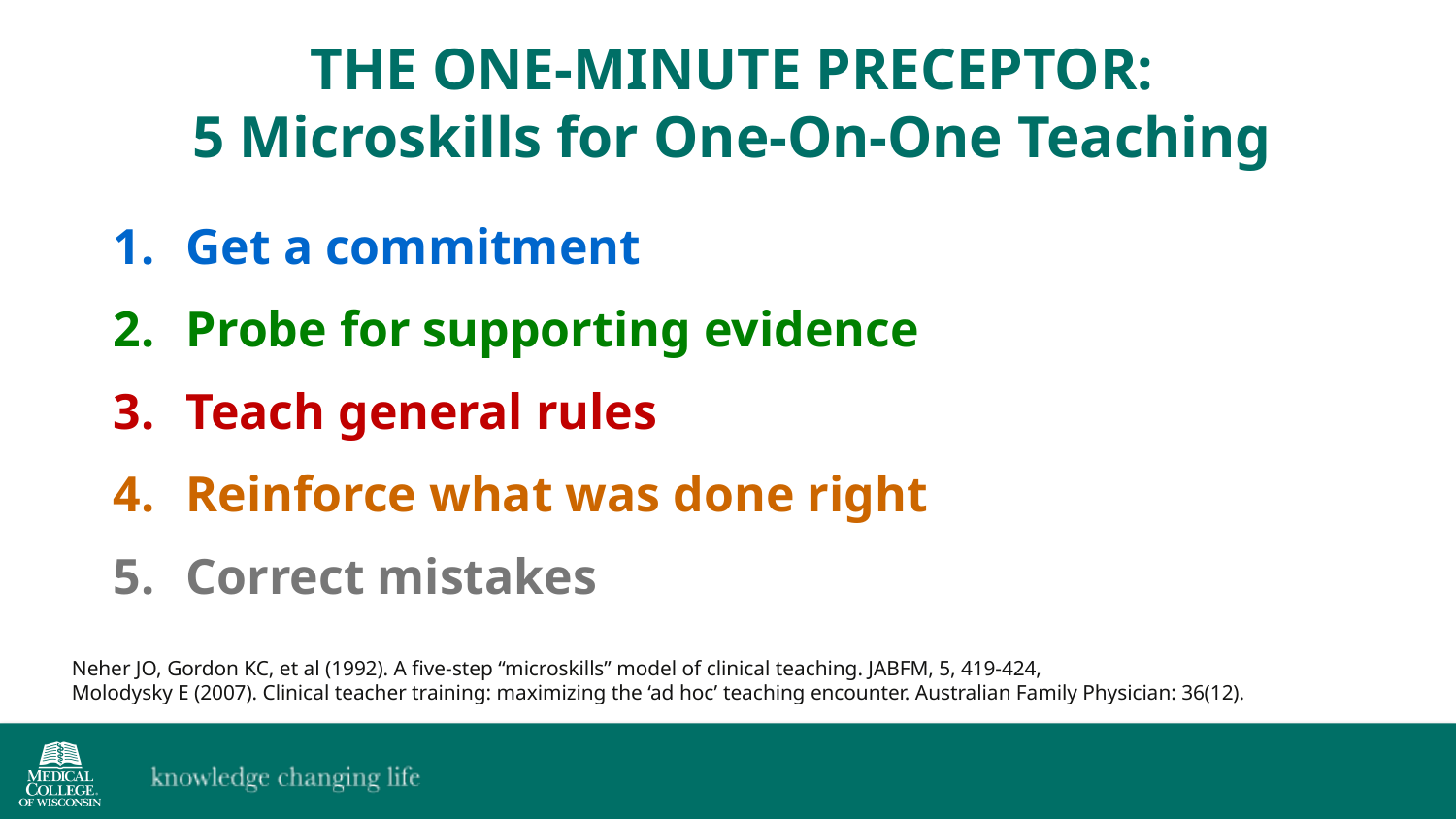

The One-Minute Preceptor:
5 Microskills for One-On-One Teaching
Get a commitment
Probe for supporting evidence
Teach general rules
Reinforce what was done right
Correct mistakes
Neher JO, Gordon KC, et al (1992). A five-step “microskills” model of clinical teaching. JABFM, 5, 419-424,
Molodysky E (2007). Clinical teacher training: maximizing the ‘ad hoc’ teaching encounter. Australian Family Physician: 36(12).

## Slide 24
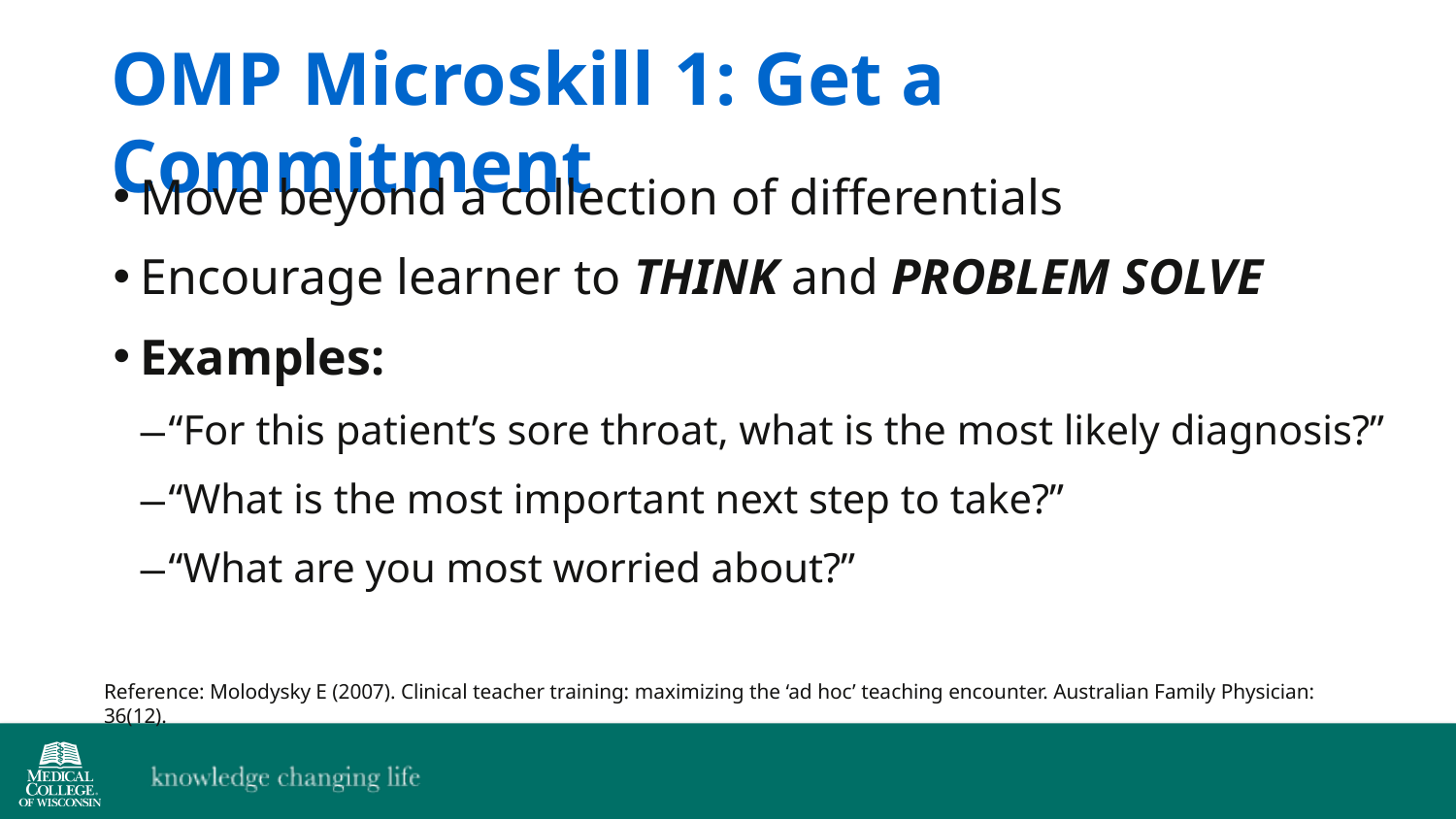

OMP Microskill 1: Get a Commitment
Move beyond a collection of differentials
Encourage learner to THINK and PROBLEM SOLVE
Examples:
“For this patient’s sore throat, what is the most likely diagnosis?”
“What is the most important next step to take?”
“What are you most worried about?”
Reference: Molodysky E (2007). Clinical teacher training: maximizing the ‘ad hoc’ teaching encounter. Australian Family Physician: 36(12).

## Slide 25
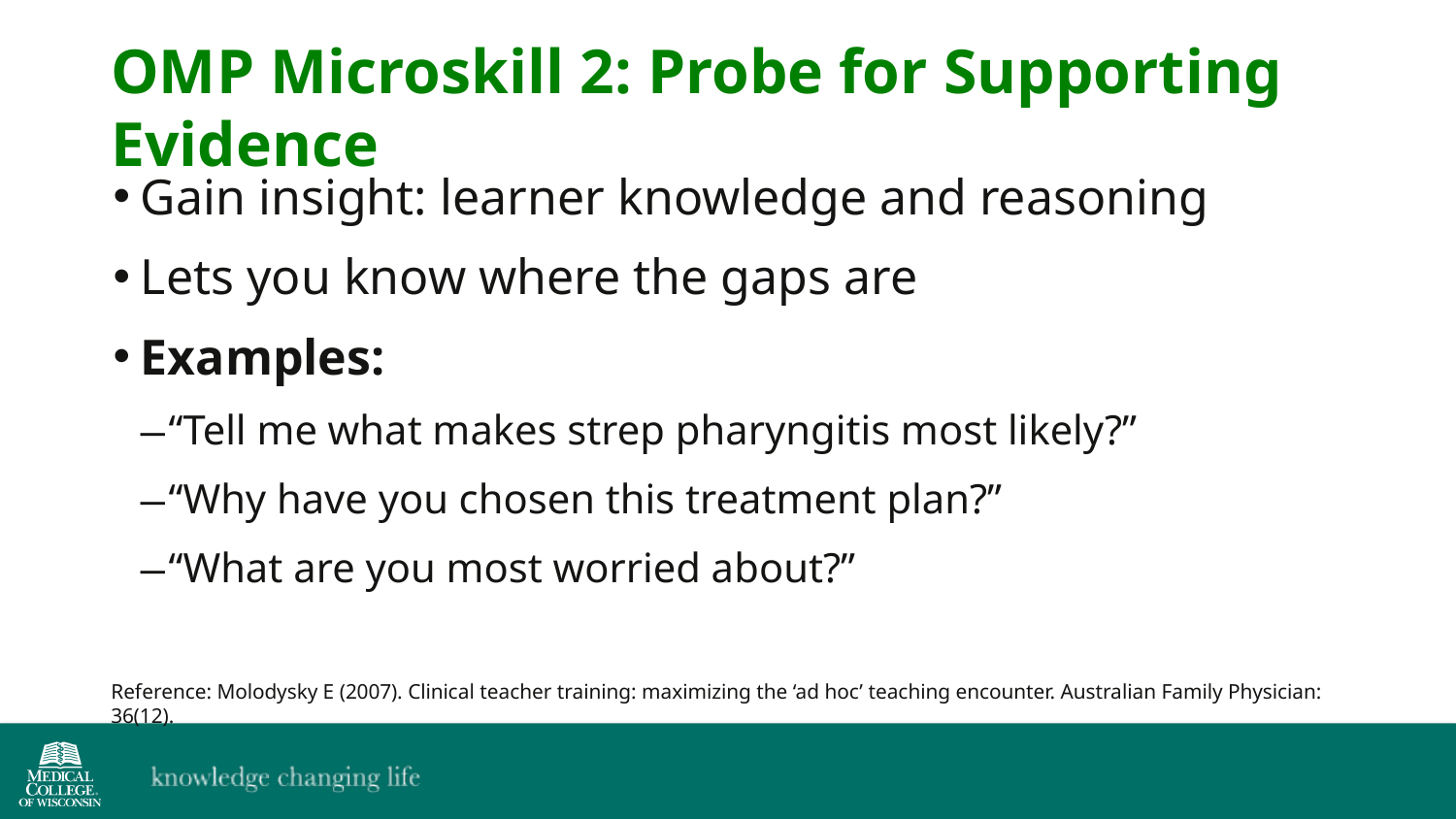

OMP Microskill 2: Probe for Supporting Evidence
Gain insight: learner knowledge and reasoning
Lets you know where the gaps are
Examples:
“Tell me what makes strep pharyngitis most likely?”
“Why have you chosen this treatment plan?”
“What are you most worried about?”
Reference: Molodysky E (2007). Clinical teacher training: maximizing the ‘ad hoc’ teaching encounter. Australian Family Physician: 36(12).

## Slide 26
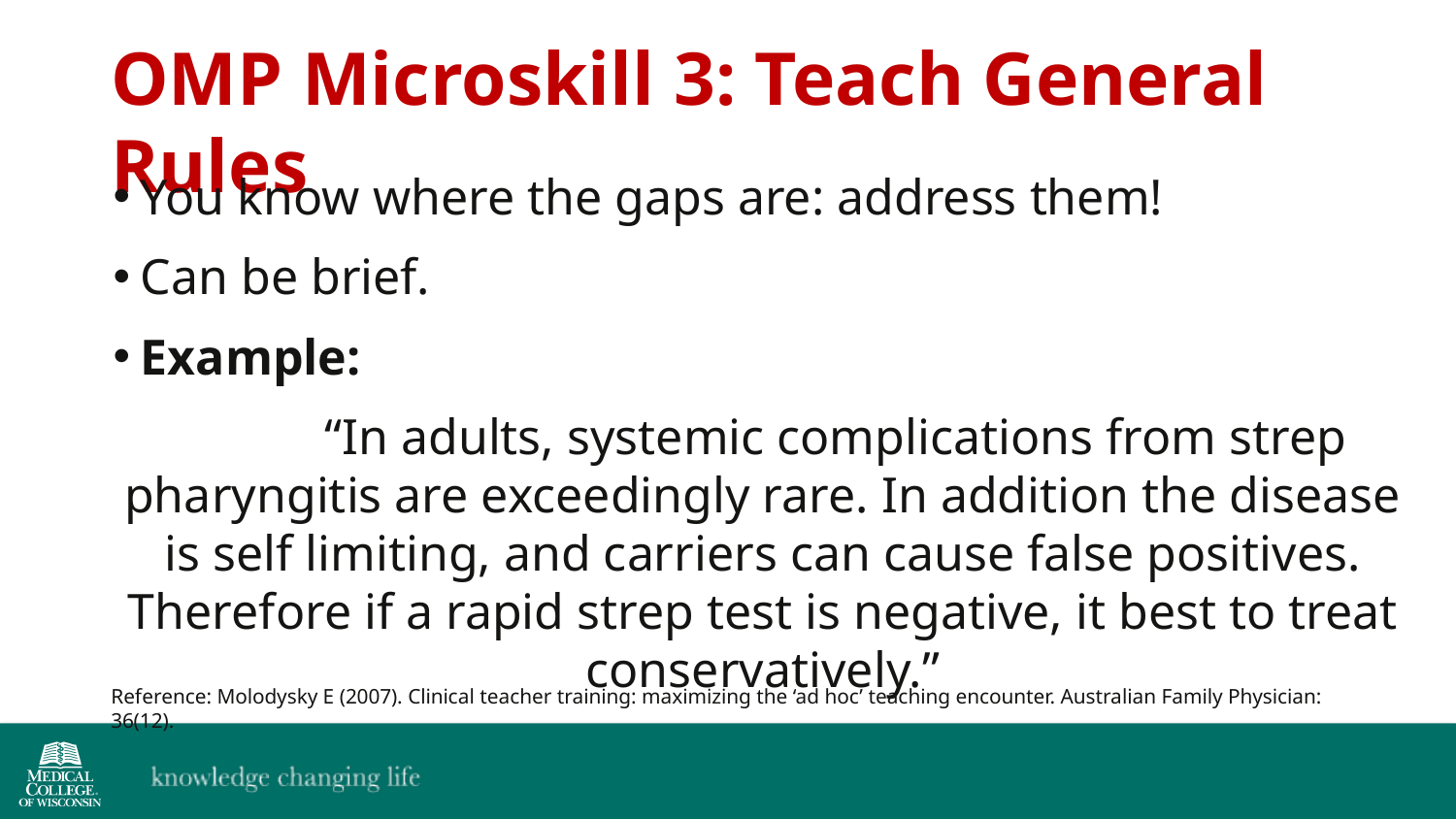

OMP Microskill 3: Teach General Rules
You know where the gaps are: address them!
Can be brief.
Example:
	“In adults, systemic complications from strep pharyngitis are exceedingly rare. In addition the disease is self limiting, and carriers can cause false positives. Therefore if a rapid strep test is negative, it best to treat conservatively.”
Reference: Molodysky E (2007). Clinical teacher training: maximizing the ‘ad hoc’ teaching encounter. Australian Family Physician: 36(12).

## Slide 27
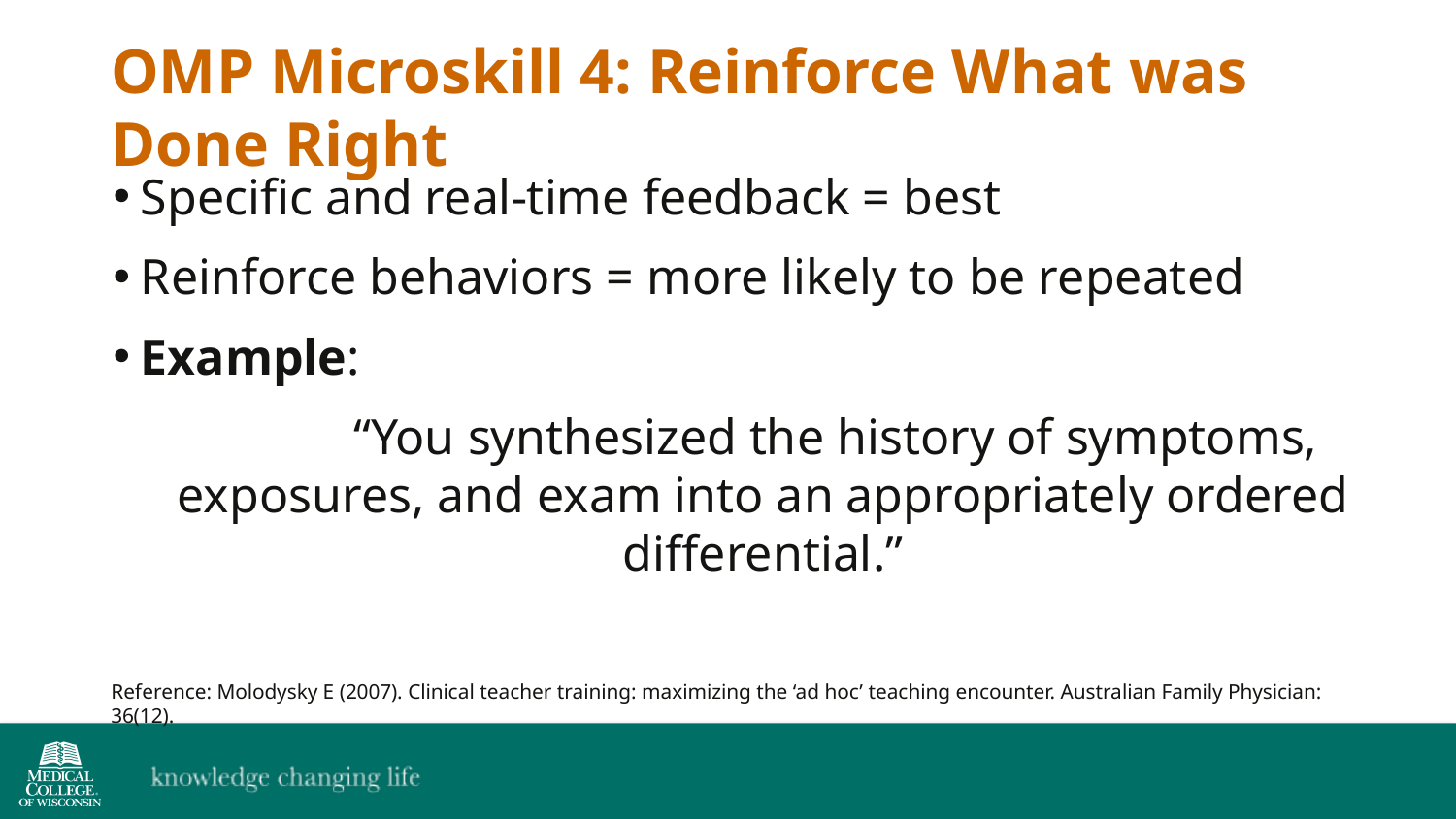

OMP Microskill 4: Reinforce What was Done Right
Specific and real-time feedback = best
Reinforce behaviors = more likely to be repeated
Example:
	“You synthesized the history of symptoms, exposures, and exam into an appropriately ordered differential.”
Reference: Molodysky E (2007). Clinical teacher training: maximizing the ‘ad hoc’ teaching encounter. Australian Family Physician: 36(12).

## Slide 28
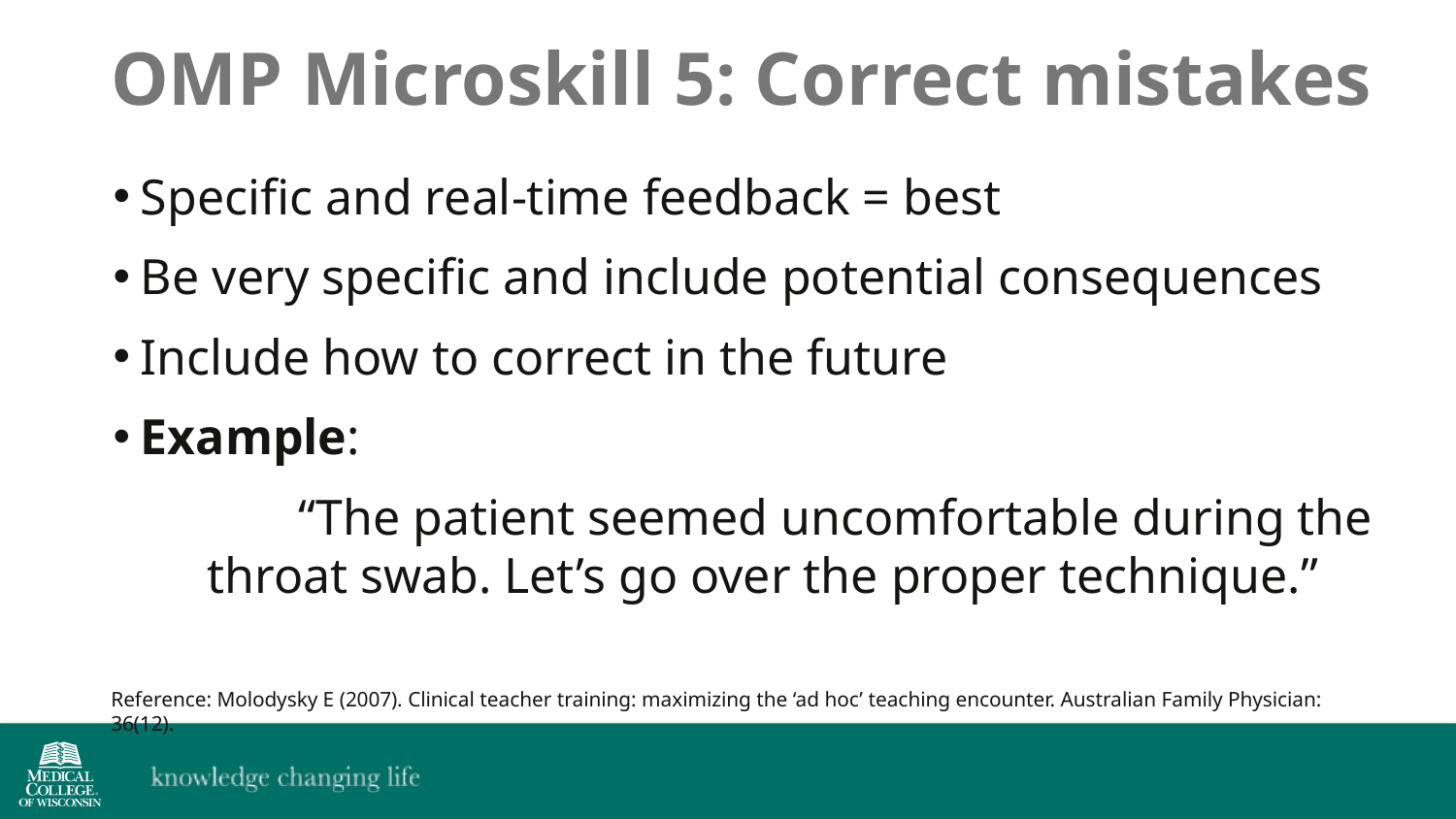

OMP Microskill 5: Correct mistakes
Specific and real-time feedback = best
Be very specific and include potential consequences
Include how to correct in the future
Example:
	“The patient seemed uncomfortable during the throat swab. Let’s go over the proper technique.”
Reference: Molodysky E (2007). Clinical teacher training: maximizing the ‘ad hoc’ teaching encounter. Australian Family Physician: 36(12).

## Slide 29
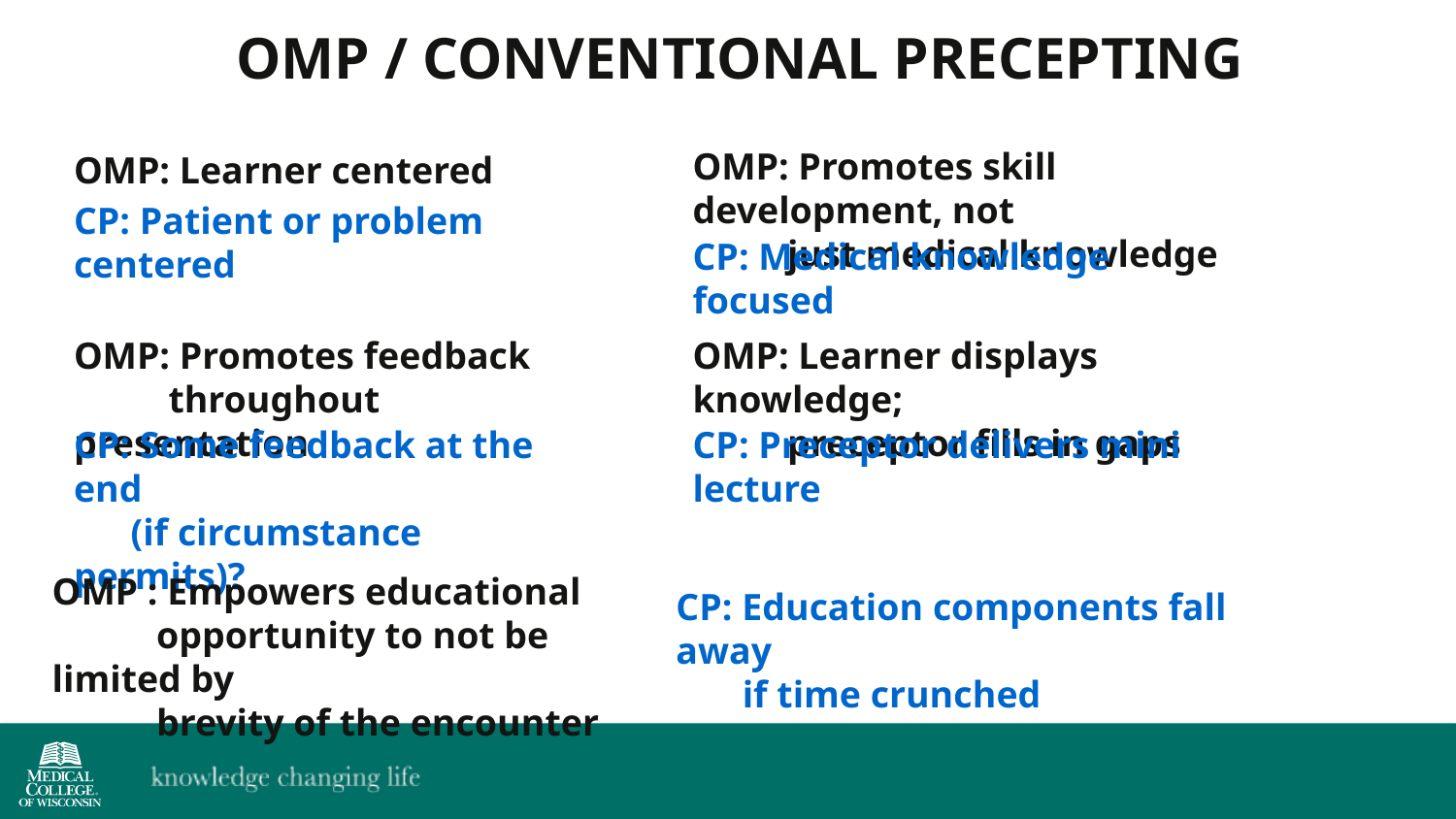

OMP / Conventional Precepting
OMP: Promotes skill development, not
 just medical knowledge
OMP: Learner centered
CP: Patient or problem centered
CP: Medical knowledge focused
OMP: Promotes feedback
 throughout presentation
OMP: Learner displays knowledge;
 preceptor fills in gaps
CP: Some feedback at the end
 (if circumstance permits)?
CP: Preceptor delivers mini lecture
OMP : Empowers educational
 opportunity to not be limited by
 brevity of the encounter
CP: Education components fall away
 if time crunched

## Slide 30
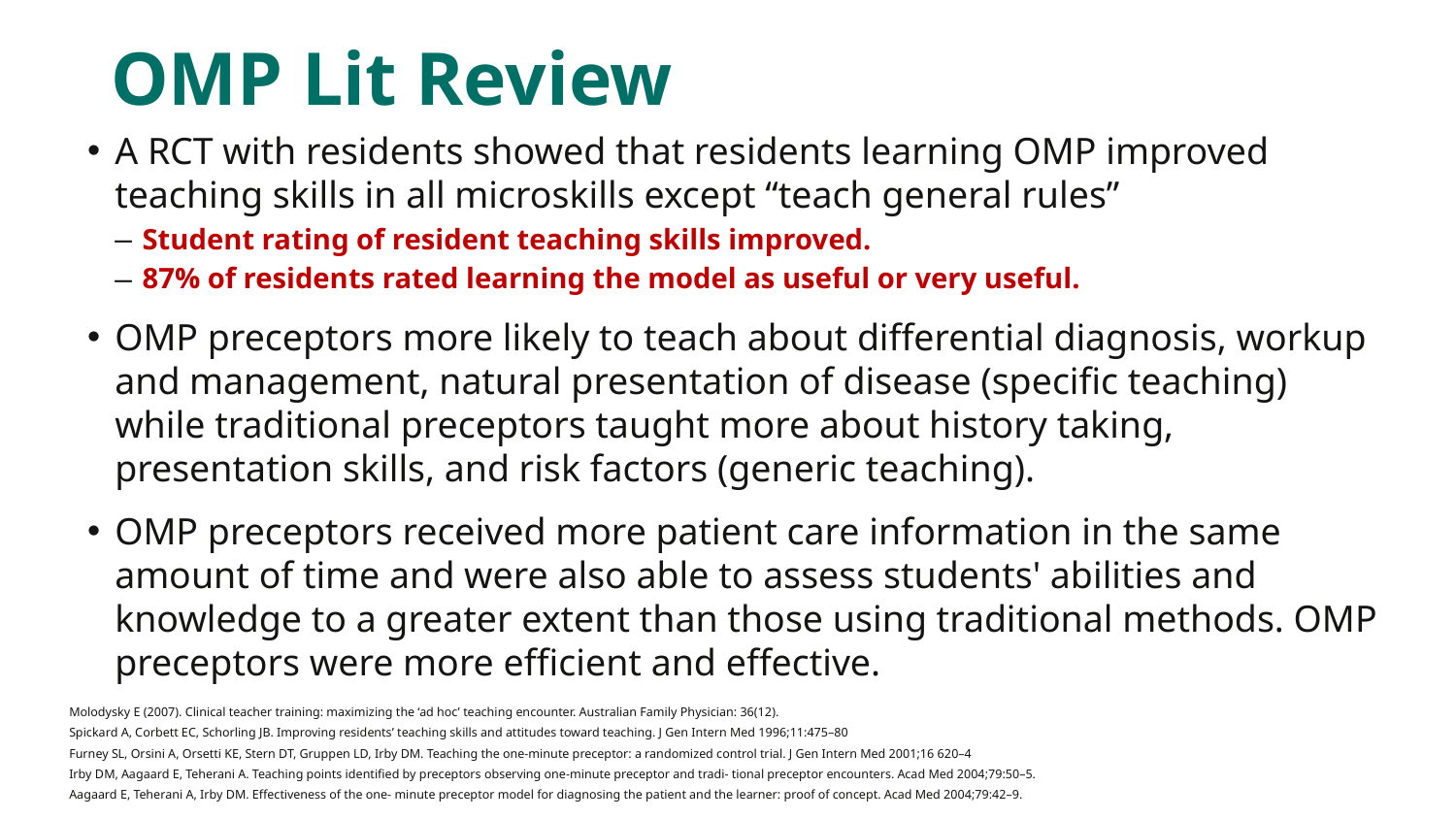

OMP Lit Review
A RCT with residents showed that residents learning OMP improved teaching skills in all microskills except “teach general rules”
Student rating of resident teaching skills improved.
87% of residents rated learning the model as useful or very useful.
OMP preceptors more likely to teach about differential diagnosis, workup and management, natural presentation of disease (specific teaching) while traditional preceptors taught more about history taking, presentation skills, and risk factors (generic teaching).
OMP preceptors received more patient care information in the same amount of time and were also able to assess students' abilities and knowledge to a greater extent than those using traditional methods. OMP preceptors were more efficient and effective.
Molodysky E (2007). Clinical teacher training: maximizing the ‘ad hoc’ teaching encounter. Australian Family Physician: 36(12).
Spickard A, Corbett EC, Schorling JB. Improving residents’ teaching skills and attitudes toward teaching. J Gen Intern Med 1996;11:475–80
Furney SL, Orsini A, Orsetti KE, Stern DT, Gruppen LD, Irby DM. Teaching the one-minute preceptor: a randomized control trial. J Gen Intern Med 2001;16 620–4
Irby DM, Aagaard E, Teherani A. Teaching points identified by preceptors observing one-minute preceptor and tradi- tional preceptor encounters. Acad Med 2004;79:50–5.
Aagaard E, Teherani A, Irby DM. Effectiveness of the one- minute preceptor model for diagnosing the patient and the learner: proof of concept. Acad Med 2004;79:42–9.

## Slide 31
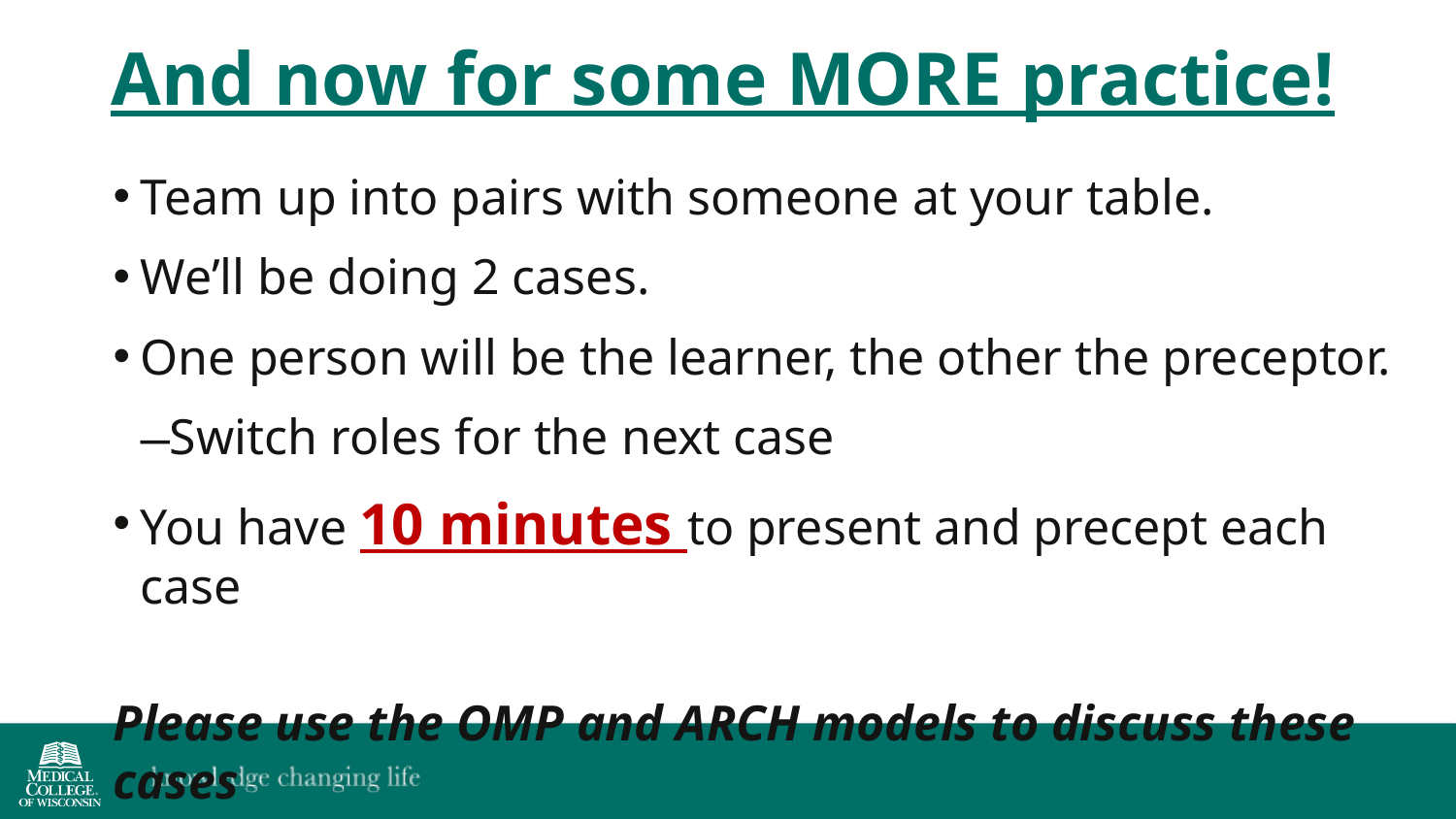

And now for some MORE practice!
Team up into pairs with someone at your table.
We’ll be doing 2 cases.
One person will be the learner, the other the preceptor.
Switch roles for the next case
You have 10 minutes to present and precept each case
Please use the OMP and ARCH models to discuss these cases

## Slide 32
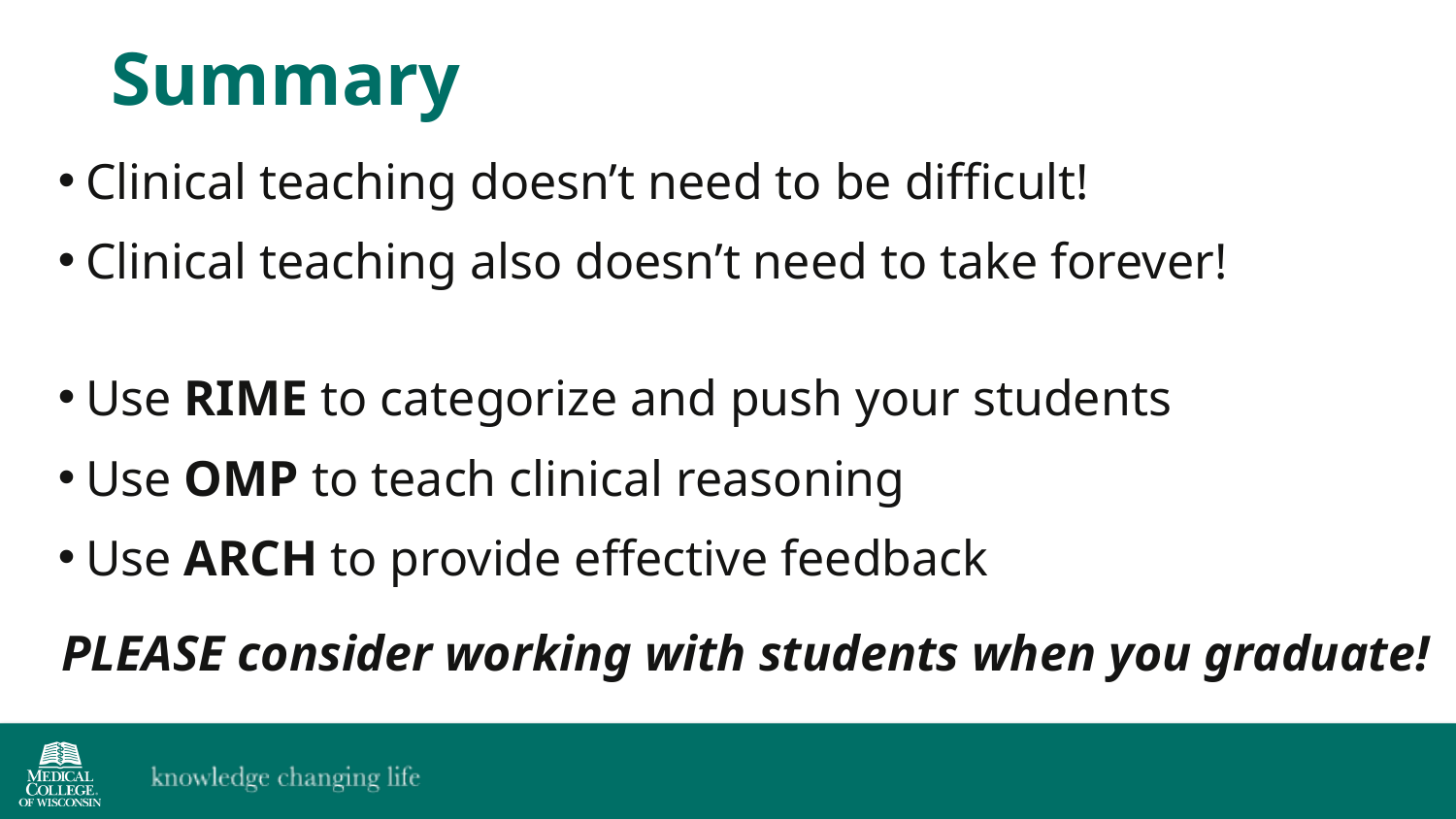

Summary
Clinical teaching doesn’t need to be difficult!
Clinical teaching also doesn’t need to take forever!
Use RIME to categorize and push your students
Use OMP to teach clinical reasoning
Use ARCH to provide effective feedback
PLEASE consider working with students when you graduate!

## Slide 33
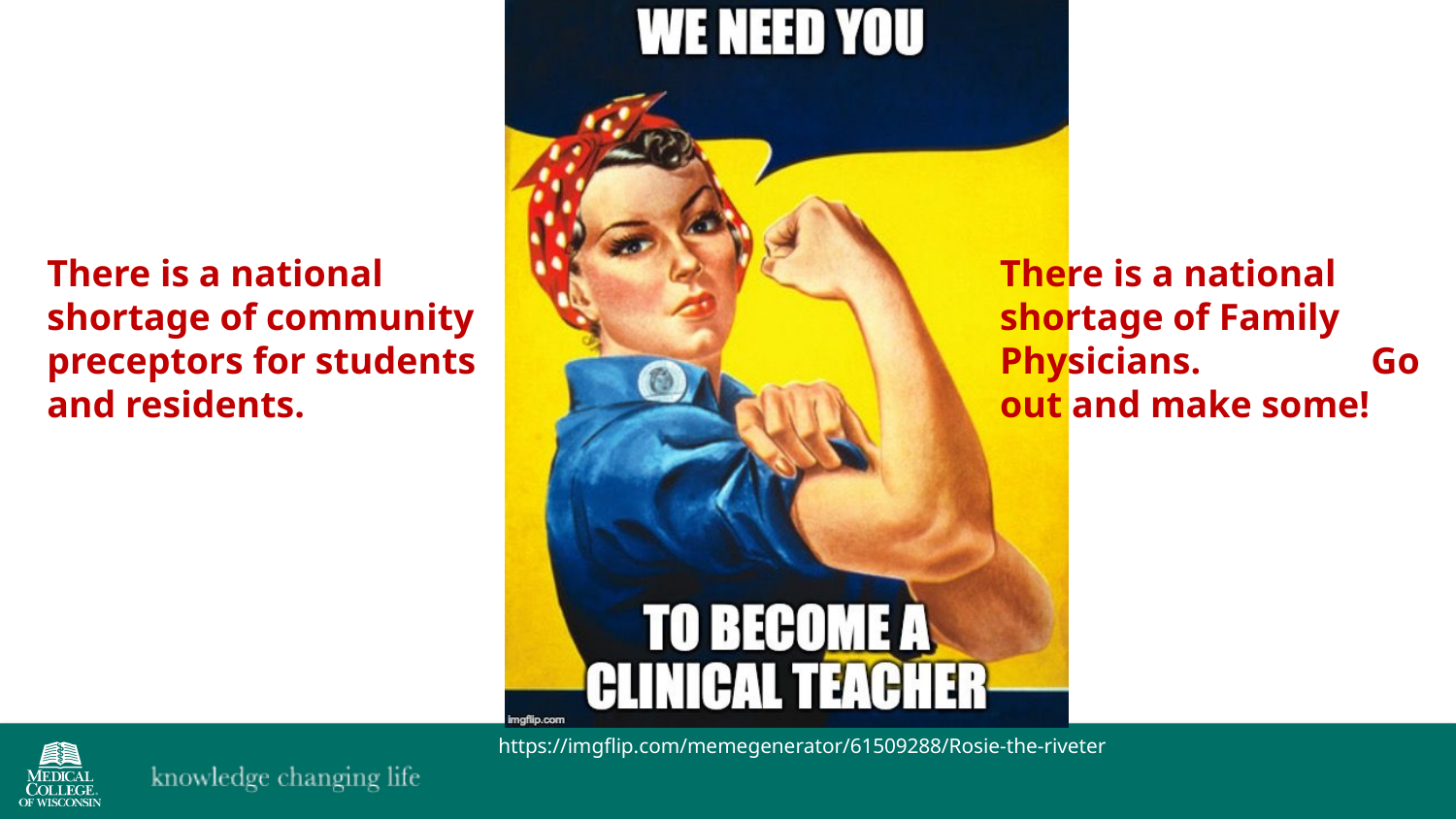

There is a national shortage of community preceptors for students and residents.
There is a national shortage of Family Physicians. Go out and make some!
https://imgflip.com/memegenerator/61509288/Rosie-the-riveter
