# Supplementary material for: Outpatient Teaching and Feedback Skills Workshop for Resident Physicians
Source: MedEdPORTAL. 2020 Jul 31;16:10930. doi: 10.15766/mep_2374-8265.10930 (PMC7394347; doi:10.15766/mep_2374-8265.10930)
Supplement: Supplementary file 1 — ARCH, RIME, and OMP Training Materials.pptxPocket Teaching Guide.docxRIME Role-Play Case Studies.docxOMP Role-Play Case Studies.docxPre- and Posttest.docx [file mep_2374-8265.10930-s001.zip › B. Pocket Teaching Guide.docx]

**Pocket Teaching Guide**

One Minute Preceptor

Goal: Teach clinical reasoning quickly!

1. Get a commitment
2. Probe for supporting evidence
3. Teach general rules
4. Reinforce what was done correctly
5. Correct Mistakes

RIME Model

Goal: Help the learner advance to the next stage!

1. Observer
2. Reporter
3. Interpreter
4. Manager
5. Educator

ARCH Feedback Model

Goal: Provide quick, effective feedback and guidance!

1. Ask for self-assessment
2. Reinforce what is being done well
3. Correct what needs improvement
4. Help with plans for improvement
