# Supplementary material for: Outpatient Teaching and Feedback Skills Workshop for Resident Physicians
Source: MedEdPORTAL. 2020 Jul 31;16:10930. doi: 10.15766/mep_2374-8265.10930 (PMC7394347; doi:10.15766/mep_2374-8265.10930)
Supplement: Supplementary file 1 — ARCH, RIME, and OMP Training Materials.pptxPocket Teaching Guide.docxRIME Role-Play Case Studies.docxOMP Role-Play Case Studies.docxPre- and Posttest.docx [file mep_2374-8265.10930-s001.zip › C. RIME Role-Play Case Studies.docx]

RIME case #1-Preceptor

Chief complaint: pain with urination

Prompt: use the RIME model to help the student through their presentation, they have been instructed to stop at a certain point. Determine which stage of the model they are currently exhibiting (ie, observer, reporter, interpreter, manager, or educator). Target your teaching to their current level, and focus this teaching to help coach them into the next level (ie from interpreter to manager). Use your pocket card to guide your teaching approach.

Feedback: use the ARCH model to give feedback to the student.

After finishing the case, compare notes with your colleague and see if you agree on the level of RIME learner capability this case represents.

RIME case #1-Student

Present the following case to your preceptor, as you would in a staffing session in clinic. Follow the prompts. You are a good reporter, working on your interpretation.

Chief complaint: pain with urination

HPI:

- 24 year old Female, no significant PMH
- 2 days dysuria, frequency, urgency
- Urine appears bloody
- Mild lower abdominal discomfort, nausea; no vomiting
- No flank pain
- No fevers or chills
- No change in vaginal discharge

Physical exam:

Vitals: temp 98.9, HR 74, RR 16, BP 102/64

Gen: alert, no distress

CV: normal rate, regular rhythm; no murmurs/rubs/gallops

Abd: soft, mild tenderness in suprapubic region, no guarding or rebound; no CVA tenderness

Labs:

UA: 3+ bacteria, large leukocyte esterase, + nitrites, small blood

Assessment/plan: **at this point, stop.** The preceptor needs to prompt you for your differential, and perhaps your management. You may give 1 diagnosis if you want (maybe a wrong one), but do not give a full differential. The goal is to make the preceptor guide you to a differential diagnosis (ie from a reporter into an interpreter).

Feedback: use the ARCH model to give feedback to the preceptor

After finishing the case, compare notes with your colleague and see if you agree on the stage of the RIME continuum this case represents.

RIME case #2-Preceptor

Chief complaint: Headaches

Prompt: use the RIME model to help the student through their presentation, they have been instructed to stop at a certain point. Determine which stage of the model they are currently exhibiting (ie, observer, reporter, interpreter, manager, or educator). Target your teaching to their current level, and focus this teaching to help coach them into the next level (ie from interpreter to manager). Use your pocket card to guide your teaching approach.

Feedback: use the ARCH model to give feedback to the student.

After finishing the case, compare notes with your colleague and see if you agree on the level of RIME learner capability this case represents.

RIME case #2-Student

Chief complaint: headaches

HPI:

- 34 year old female, no significant PMH
- Complains of unilateral headache, intense behind the left eye
- Becomes sensitive to light and sound during the headache
- Seems to be worse around the menstrual cycle
- Sometimes has difficulty speaking during these episodes
- Lots of stress in her life, single mom of 3 kids, no financial support.
- Working 2 jobs, gets about 5 hours of sleep max.
- Mostly eats fast food between her jobs

Physical exam:

Vitals: temp 98.9, HR 74, RR 18, BP 160/98, BMI 42

Gen: alert, no distress

CV: Regular rate and rhythm, no murmurs, rubs, gallops

Respiratory: Lungs clear to auscultation bilaterally

Neuro: Cranial nerves intact x 12. No sensory or motor deficits.

A/P: **at this point, provide a differential diagnosis and then STOP.** Do not present a plan. Your preceptor should help guide you toward a management plan (from interpreter into a manager).

*Proposed Differential: Migraine headaches, Tension Headaches, Hypertension headaches, hyperglycemia*

Feedback: use the ARCH model to give feedback to the preceptor

After finishing the case, compare notes with your colleague and see if you agree on the stage of the RIME continuum this case represents.
