# Supplementary material for: Outpatient Teaching and Feedback Skills Workshop for Resident Physicians
Source: MedEdPORTAL. 2020 Jul 31;16:10930. doi: 10.15766/mep_2374-8265.10930 (PMC7394347; doi:10.15766/mep_2374-8265.10930)
Supplement: Supplementary file 1 — ARCH, RIME, and OMP Training Materials.pptxPocket Teaching Guide.docxRIME Role-Play Case Studies.docxOMP Role-Play Case Studies.docxPre- and Posttest.docx [file mep_2374-8265.10930-s001.zip › D. OMP Role-Play Case Studies.docx]

**OMP Case 1 - Medical student prompt**

(Read the case and present it to your colleague as you would in a precepting session)

**HPI:** Patient is a 52-year-old Caucasian male with past medical history significant for diabetes, hypertension, neuropathy that presents to the office today for diabetes follow-up. Patient reports that he has worsening neuropathy in his toes that have now spread to ankles bilaterally. Patient also reports tingling in his fingertips. Notes increased thirst and increased urinary frequency. Patient reports eating a high carbohydrate diet and exercises “at times”. Patent has not seen an eye doctor or podiatrist or dentist “in a long time”.

**Medications:** Metformin 1000mg twice daily, Glyburide 5mg daily, Lisinopril 20 daily,

Admits he takes his medication “when he has time” and he ran out several days ago.

**Social History:** Drinks 1-2 beers a couple times a week. Smokes 1-2 cigarettes a day, denies drug use. Not currently sexually active.

**Physical exam**

BMI: 30; Weight: 100 kg; Blood pressure: 147/94 mm Hg; Respiratory rate: 18 rpm; Heart rate: 99 bpm

**General exam**: Disheveled, no distress, alert male.

**Eye exam:** Pupils are equal and reactive to light. Fundal exam normal.

**Oral cavity:** Poor dentition noted with multiple cavities.

**Cardiovascular:** Regular rate and rhythm. No murmurs noted. Normal peripheral pulses

**Pulmonary:** No respiratory distress. No wheezing, rales, crackles.

**Abdominal**: Obese abdomen. Abdomen is soft, nontender, nondistended.

**Neurological:** Strength 5/5 in all extremities. Decreased sensation to vibration and has difficulty telling the difference between sharp and dull in fingertips and toes. Monofilament exam notable for decreased sensation over anterior shins bilaterally.

**Skin**: No ulcerations noted.

**Foot:** Thick, yellow toenails on both feet. No ulcers.

Point of care hemoglobin A1c: 12

**Assessment & Plan: Uncontrolled diabetes**

1. continue metformin 1000 mg twice daily
2. Discontinue glyburide 5 mg daily “because it causes weight gain”
3. Start liraglutide 5mg daily
4. Start glargine insulin 10 units nightly
5. Encourage diet rich in lean protein, vegetables and fresh fruit
6. Encourage patient to get a local gym membership and exercise 3 times a week
7. Refill all medications and encourage follow-up in 2 weeks
8. Refer to a podiatrist and ophthalmologist

After you present the above case, allow your preceptor to provide you with some guidance.

Your preceptor will ask you to do a self-assessment. Please emphasize that you are satisfied with your care of this patient, in particular the plans you have made to improve control of his diabetes.

**OMP Case 1 - Attending prompt**

You know this patient’s social history well including that he has significant financial barriers which have contributed to his lack of adherence to plans in the past. His meals consistently come from a food pantry at a local church, or other affordable and often carbohydrate-rich sources which do not require him to cook.

It is clear to you that the student’s lack of exploring this patient’s social context and barriers to care have impacted this visit’s chance of success. It is very unlikely that this patient can afford fresh produce or a gym membership. You also know that glargine and liraglutide are very expensive and not likely covered by patient’s insurance, which may result in further delays in access to care. This patient may benefit from cheaper insulin like NPH, medication affordability resources such as GoodRX or community charity pharmacy programs, more accessible exercise and diet recommendations, and further assessment of social barriers to care and interventions to mitigate them.

**Please use the one-minute preceptor model to discuss this case with your student**

**Once the case is done, please use the ARCH feedback model to coach your student**

**OMP Case 2 – Student Prompt**

(Read the case below and present it to your attending in your own words)

Chief Complaint: Chest pain

**HPI:** Your patient complains of chest pain. Position changes, coughing, and deep breathing provoke the pain, and nothing has palliated it; it feels sharp; it involves central chest and and radiates to diffusely involve the chest at times; it is 9 out of 10 in severity; it has been worsening over the past 3 days over which time the patient has noticed a worsening cough.

**PMH:** Obesity and Hypertension

**Medications:** Hydrochlorothiazide 25mg daily

**Family history:** Grandmother and Aunt have “bad hearts”.

**Social history:** Occasional alcohol use on the weekends. Denies other drug use. Lives at home with her husband. She is a child care worker.

**Vitals:** Temperature: 98.0 degrees Fahrenheit; Heart rate: 85 bpm; Blood Pressure: 135/85 mmHg; Respiratory Rate: 18 rpm.

**Exam:**

**General exam**: Comfortable in the exam room.

**Cardiovascular:** Regular rate and rhythm. No murmurs, rubs or gallops. 2+ peripheral pulses.

**Respiratory:** Lungs clear to auscultation bilaterally. Taking deep breaths causes patient to grimace and grasp her chest.

**Abdominal:** Soft, non-distended, non-tender in all quadrants. Normal bowel sounds.

**Musculoskeletal:** Strength intact. Tenderness to palpation over sternum.

**Neurological:** 2+ peripheral reflexes. Cranial nerves grossly intact. No focal deficits.

**Assessment & Plan**: Your differential includes Acute Coronary Syndrome (ACS), pneumonia, and costochondritis. You are uncertain which it is. You have informed the patient she “might be having a heart attack but you have to ask your preceptor first”.

**Please stop after presenting your differential. Allow your attending to coach you through the rest of the encounter.**

*When your preceptor asks you to commit to the most likely diagnosis, choose ACS.*

*List obesity and family history as supporting evidence.*

**Instructions for feedback:**

Your preceptor will give you feedback using the ARCH model

*When asked: say that you are disappointed in your judgement and feel like you have really let your patient down.*

**OMP Case 2 – Attending Prompt**

Listen to the student’s presentation and guide them using the one-minute preceptor model of teaching. After the student gives their differential, ask them to commit to what they think is the most likely diagnosis.

When the case concludes, please use the ARCH feedback model to discuss the case with your student
