# Supplementary material for: Outpatient Teaching and Feedback Skills Workshop for Resident Physicians
Source: MedEdPORTAL. 2020 Jul 31;16:10930. doi: 10.15766/mep_2374-8265.10930 (PMC7394347; doi:10.15766/mep_2374-8265.10930)
Supplement: Supplementary file 1 — ARCH, RIME, and OMP Training Materials.pptxPocket Teaching Guide.docxRIME Role-Play Case Studies.docxOMP Role-Play Case Studies.docxPre- and Posttest.docx [file mep_2374-8265.10930-s001.zip › E. Pre- and Posttest.docx]

MCW residents as teachers pre/post quiz

Please self-assess your views on point of care teaching and feedback styles in a busy clinic

1. Teaching in clinic is too time consuming. I’m unlikely to do it in my future career
2. I’m unfamiliar with the various teaching techniques but I might do it in the future
3. I have some idea of basic clinical teaching concepts. I’m unsure if I’ll do it in the future
4. I have a good grasp of basic clinical teaching concepts. I’ll probably do it in the future
5. I’ve mastered clinical teaching concepts and plan to work with medical students in clinic

The first (and most important) concept step in the One Minute Preceptor is:

1. Give a mini lecture on the case
2. Get a commitment from the learner
3. Correct learner’s mistakes
4. Ask increasingly difficult questions until the learner gets one wrong

A learner has just reports a patient’s HPI and exam but did not give a diagnosis or suggest a management plan. Using the RIME model, what is the best way to help them along?

1. Ask them to give you a 5 minute talk on the case
2. Get them to commit to a management plan
3. Get them to commit to a diagnosis
4. Give a mini lecture on what you think the diagnosis and management plan should be

A learner has just finished their clinical rotation with you. Using the ARCH model, what is the first step in providing feedback?

1. Provide your assessment of the learner’s abilities
2. Propose an improvement plan for your learner
3. Ask the learner to give you a self assessment
4. Provide a comprehensive list of all the learners knowledge gaps and deficienceis
